# Endosymbiotic adaptations in three new bacterial species associated with *Dictyostelium discoideum*: *Paraburkholderia agricolaris* sp. nov., *Paraburkholderia hayleyella* sp. nov., and *Paraburkholderia bonniea* sp. nov

**DOI:** 10.7717/peerj.9151

**Published:** 2020-05-22

**Authors:** Debra A. Brock, Suegene Noh, Alicia N.M. Hubert, Tamara S. Haselkorn, Susanne DiSalvo, Melanie K. Suess, Alexander S. Bradley, Mahboubeh Tavakoli-Nezhad, Katherine S. Geist, David C. Queller, Joan E. Strassmann

**Affiliations:** 1 Department of Biology, Washington University in St. Louis, St Louis, MO, United States of America; 2 Department of Biology, Colby College, Waterville, ME, United States of America; 3 Department of Biology, University of Central Arkansas, Conway, AR, United States of America; 4 Department of Biological Sciences, Southern Illinois University at Edwardsville, Edwardsville, IL, United States of America; 5 Department of Earth and Planetary Sciences, Washington University in St. Louis, St Louis, MO, United States of America; 6 Department of Earth and Planetary Sciences, Division of Biology and Biomedical Sciences, Washington University in St. Louis, St Louis, MO, United States of America

**Keywords:** Symbiosis, Mutualism, Social amoebae, Dictyostelium, Paraburkholderia

## Abstract

Here we give names to three new species of *Paraburkholderia* that can remain in symbiosis indefinitely in the spores of a soil dwelling eukaryote, *Dictyostelium discoideum*. The new species *P. agricolaris* sp. nov.*, P. hayleyella* sp. nov.*,* and *P. bonniea* sp. nov*.* are widespread across the eastern USA and were isolated as internal symbionts of wild-collected *D. discoideum*. We describe these sp. nov. using several approaches. Evidence that they are each a distinct new species comes from their phylogenetic position, average nucleotide identity, genome-genome distance, carbon usage, reduced length, cooler optimal growth temperature, metabolic tests, and their previously described ability to invade *D. discoideum* amoebae and form a symbiotic relationship*.* All three of these new species facilitate the prolonged carriage of food bacteria by *D. discoideum,* though they themselves are not food. Further studies of the interactions of these three new species with *D. discoideum* should be fruitful for understanding the ecology and evolution of symbioses.

## Introduction

Eukaryote soil-dwelling amoebae are exposed to bacteria in their environments. Amoebae ingest bacteria, but some bacteria may foil their digestive systems and take up residence inside amoebae (Molmeret et al., 2005; Evstigneeva et al., 2009). Amoebae may also be penetrated by bacteria using secretion systems (Tosetti, Croxatto & Greub, 2014). Some of these bacteria evidently become permanent or semi-permanent residents (Oliver et al., 2010). In this study, we examine the characteristics of *Paraburkholderia* that have formed long-term symbiotic relationships with the social amoeba *Dictyostelium discoideum* (Brock et al., 2011; DiSalvo et al., 2015; Brock et al., 2016). Based on data presented here and data previously published (Brock et al., 2011; DiSalvo et al., 2015; Brock et al., 2016; Shu et al., 2018a; Shu et al., 2018b; Haselkorn et al., 2019), we name three new species in the plant beneficial and environmental *Paraburkholderia* clade of *Burkholderia* sensu lato*.*

The genus *Burkholderia* sensu lato is comprised of over 60 species that were originally included in the genus *Pseudomonas*, but was identified as unique by Yabuuchi et al. (1992). *Burkholderia* sensu lato are diverse and include species that are adapted for life in the soil, as endosymbionts, and as pathogens for both plants and animals. Of the pathogenic bacteria, there is a group of 20 species that are together identified as the *Burkholderia cepacia* complex, which are most predominantly associated with infections that can be lethal in immunocompromised human patients, most notably, patients with cystic fibrosis (Mahenthiralingam, Urban & Goldberg, 2005; Sousa et al., 2017). Sawana, Adeolu & Gupta (2014) proposed separating the genus into two separate genera: *Burkholderia* sensu stricto*,* which contains the pathogenic organisms, and *Paraburkholderia* which contains the environmental species. Further examination of this clade reveals that this separation and reclassification may be premature because of difficulties in placing intermediate species (Vandamme et al., 2017). However, three additional genera, *Caballeronia*, *Mycetohabitans*, and *Trinickia*, have been proposed since (Dobritsa & Samadpour, 2016; Estrada-de los Santos et al., 2018) and the separation of genera within *Burkholderia* sensu lato holds up with additional genome-scale evidence (Buekes et al., 2017). Therefore, we apply the new genus name, *Paraburkholderia,* as it consistent with public databases (NCBI Genomes and Silva)*.*

To support naming new species, we examined multiple isolates of each species in several ways. We have already established that symbiont *Paraburkholderia* can facilitate secondary carriage of food bacteria in *D. discoideum.* We refer more specifically to this trait as farming, defined as the ability to carry food bacteria through all *D. discoideum* life stages from vegetative amoebae to formation of spores during starvation. When *D. discoideum* spores hatch in new favorable environments, the carried bacteria are also released to proliferate and in turn can be consumed by hatched amoebae (Brock et al., 2011; DiSalvo et al., 2015). We have shown two independent transitions by *Paraburkholderia* to a symbiotic relationship with *D. discoideum*, and the diversity and prevalence of symbiotic *Paraburkholderia* among a collection of 700 *D. discoideum* clones (Haselkorn et al., 2019). We found symbiont *Paraburkholderia* are attracted to and able to swim towards conditioned media prepared from *D. discoideum* amoebae: an ability important for horizontal transfer in facultative symbioses (Shu et al., 2018a). We have described native and naïve host fitness consequences to colonization by symbiont *Paraburkholderia*, and have visualized symbiont location in all host life stages (Shu et al., 2018b). Now, we place the *Paraburkholderia* isolates in a phylogeny with other *Burkholderia* species using whole genome sequencing data. Pairwise comparisons of Average Nucleotide Identity (ANI) and Genome-Genome Distance (GGD) of the three isolates to each other and to closely related *Paraburkholderia* indicate that each is a separate species. To describe and discriminate among these three *Paraburkholderia* sp. nov., we examined carbon usage using a suite of possible carbon food sources, performed fatty acid analysis and other metabolic tests, measured the length of the bacterial cells, and investigated optimal growing temperatures. Based on these data, we name 3 new species: *Paraburkholderia agricolaris* sp. nov., *Paraburkholderia hayleyella* sp. nov., and *Paraburkholderia bonniea* sp. nov.

## Material and Methods

### Bacteria isolates

**Figure 1:**
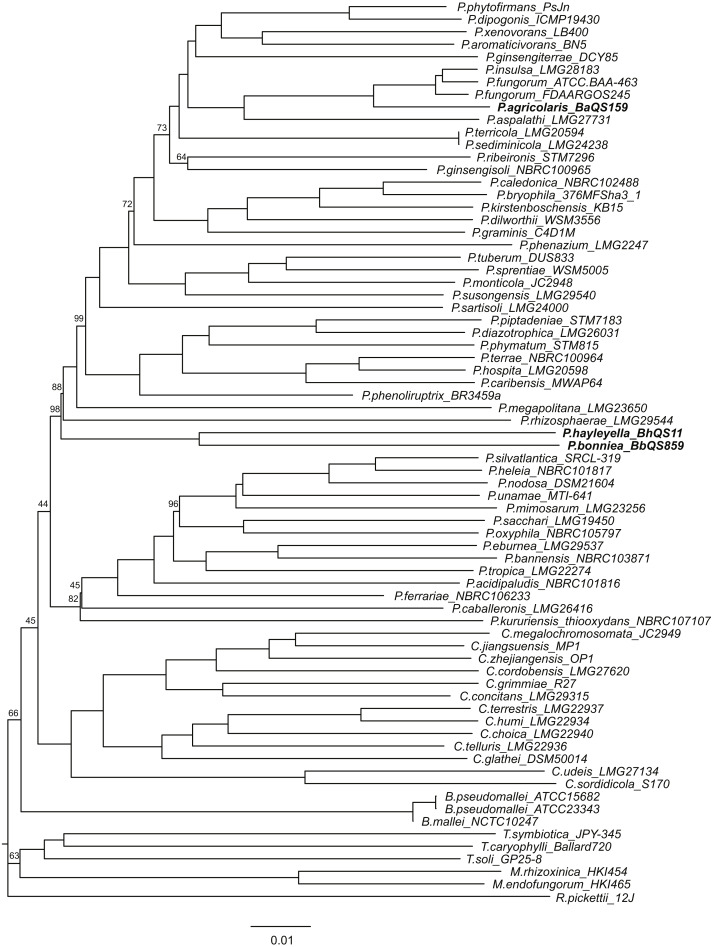
Whole genome phylogeny including three new non-pathogenic *Paraburkholderia* species found in association with *D. discoideum* and *Burkholderia* sensu lato species. We estimated the phylogenetic relationships of *D. discoideum*-associated symbionts (in bold) with 70 known species *Burkholderia* sensu lato and an outgroup species *Ralstonia pickettii*. We used k-mer frequencies (*k* = 43) of each genome and created an alignment and assembly-free distance tree. We ran 100 nonparametric bootstraps by resampling 1/k of the rows of the shared k-mer table. Any bootstrap values smaller than 100% are shown on the tree, and all other bootstrap values were fully supported.

We isolated the wild *Paraburkholderia* symbiont strains used in this study from *D. discoideum* in the Queller and Strassmann (QS) *D. discoideum* collection. See Table S1 for *D. discoideum* clones used, associated *Paraburkholderia* sp. nov., collection locations including GPS coordinates, and *Paraburkholderia* type strains used in experimental assays. We previously isolated *Paraburkholderia* strains BhQS11, BhQS21, BhQS22, BhQS155, and BaQS159 (Brock et al., 2011) and *Paraburkholderia* strains BaQS70, and BaQS175 (DiSalvo et al., 2015). *Paraburkholderia* strains BaQS31, BhQS46, BhQS115, BaQS317, BbQS433, BhQS530, BbQS859, BaQS983, and BaQS1007 are new isolates from the QS *D. discoideum* collection. All strains are stored in a sterile 20% glycerol solution at −80 °C and subcultured on SM/5 agar plates (2 g glucose, 2 g Oxoid bactopeptone, 2 g Oxoid yeast extract, 0.2 g MgSO_4_, 1.9 g KH_2_PO_4_, 1 g K_2_HPO_4_, and 15.5 g agar per liter DDH_2_O) at 22 °C. To propagate bacteria from frozen stocks for experimental assays, we plated on SM/5 nutrient agar plates and grew them at 22 °C to stationary phase. We compared patterns of carbon usage, cell length, metabolic tests, and optimal growth temperatures in representative strains of *P. agricolaris*, *P. hayleyella* and P*. bonniea* (see Table S1 for specific strains). We chose the relatively better studied members of the genus *Paraburkholderia fungorum* ATCC BAA-463, *Paraburkholderia phymatum* STM-815, and *Paraburkholderia xenovorans* LB400 for use as our *Paraburkholderia* reference control strains for experimental assays. *P. fungorum* ATCC BAA-463 is one of the closest relatives to *P. agricolaris* BaQS159 type strain. However, *P. hayleyella* BhQS11 and *P. bonniea* BbQS859 type strains are each other’s closest relatives and there are no naturally closely related type strains for comparison (Fig. 1). We included *P. phymatum* STM815 and *P. xenovorans* LB400 for comparison although these strains are equally distantly related to *P. hayleyella* BhQS11 and *P. bonniea* BbQS859. *P. xenovorans* LB400 is more closely related to *P. agricolaris* BaQS159 than the other representative strains except for *P. fungorum* ATCC BAA-483.

### Phylogenomic analyses

We isolated high-quality genomic DNA individual strains grown on SM/5 nutrient agar media using Qiagen Genomic tips (20/G). All subsequent library preparation and sequencing were performed at the University of Washington PacBio Sequencing Services. Standard PacBio sequencing libraries were prepared and sequenced on a PacBio SMRT II platform. Draft genome assemblies for BaQS159 (122× coverage) and BbQS859 (162× coverage) were analyzed via HGAP2 SmrtPipe version 1.87.139483. The draft genome assembly (243× coverage) for strain BhQS11 was previously sequenced by Duke University’s Center for Genomic and Computational Biology and analyzed via SmrtPipe version 1.85.133289. These genomes are available through NCBI Genomes and the raw sequence data are available through NCBI SRA (BioProject PRJNA480875).

We provide independent genomic evidence for phylogenetic placement of the proposed new species beyond previous multi-locus sequence typing (MLST) and partial 16S rRNA analyses (Haselkorn et al., 2019; DiSalvo et al., 2015). First, we applied alignment-free phylogenetic tree construction with our three type genome assemblies and 70 additional genomes and genome assemblies. Alignment-free or assembly-free methods that use *k*-mer (DNA words of length *k*) frequencies are increasingly available for many types of analyses that leverage next-generation sequencing data, including phylogenetic reconstruction (Zielezinski et al., 2017). Alignment-free methods provide a scalable alternative to single gene (e.g., 16S) phylogenies inferred from multiple sequence alignment. They are relatively sensitive to sequence divergence yet robust against common features of microbial genome evolution such as differences in GC content and the presence of genome rearrangements (Chan et al., 2014; Bernard, Chan & Ragan, 2016). We combined our draft assemblies with publicly available genome sequencing data that include both finished genomes and draft assemblies from an additional 47 *Paraburkholderia*, 14 *Caballeronia*, 3 *Trinickia*, 2 *Mycetohabitans*, 3 *Burkholderia sensu stricto,* and one outgroup *Ralstonia* taxa available through NCBI Genomes and JGI Integrated Microbial Genomes databases (Table S2). We estimated phylogenies using different k-mer sizes, *k* = 25, 31, 37, 43, 47, 53, and 55 with AAF (Assembly and Alignment-Free) version 20171218 (Fan et al., 2015). The number of potential *k*-mers in similar *k*-mer-based analyses increases by a factor of 4 as *k*-mer size increases. The appropriate size of *k*-mer to adopt is thus a balance between information content (larger k-mers contain more information) and computational efficiency (smaller *k*-mers require less computation and memory). The topologies of these trees stabilized at *k* = 43, therefore we based subsequent analyses on this tree. Next, we ran 100 nonparametric bootstraps and resampled 1/*k* rows from the shared *k*-mer table as described by the developer of this software (https://github.com/fanhuan/AAF; accessed Nov 2019). As an additional check, we accessed the Type (Strain) Genome Server v147 (https://tygs.dsmz.de/; accessed May 2019) to check if our choice of *Paraburkholderia* species for comparison were consistent with Genome BLAST Distance Phylogeny methods using either 16S rRNA sequences only or whole genome sequences (Meier-Kolthoff & Göker, 2019).

We also generated a full-length 16S small subunit ribosomal RNA alignment and phylogeny. Full-length 16S sequences for type strains of *Paraburkholderia* and *Caballeronia*, as well as those for *Burkholderia mallei*, *B. pseudomallei*, *Robbsia andropogonis* (mislabeled as *P. andropogonis* in the database), and *Ralstonia pickettii* were downloaded from SILVA (Quast et al., 2013; accessed Nov 2019). We only used sequences with sequence quality above 80% and included all available copies of 16S sequences from each species and strain (Klappenbach, Dunbar & Schmidt, 2000) because 16S intragenomic copy number can also taxonomically informative (Kembel et al., 2012; Ibal et al., 2019). We used SSU-ALIGN version 0.1.1 (Nawrocki, 2009) to align these sequences in a secondary structure-aware manner with the 16S sequences from our three type genomes and to trim this alignment. We used FastTree version 2.1.10 to generate an approximately-Maximum-Likelihood tree from this alignment (Price, Dehal & Arkin, 2010).

Prokaryotic species delineation was traditionally done using DNA-DNA hybridization but modern alternatives include Average Nucleotide Identity (ANI) (Konstantinidis & Tiedje, 2005; Konstantinidis, Ramette & Tiedje, 2006) and Genome-Genome Distance (GGD) (Meier-Kolthoff et al., 2014). Both methods were developed to take advantage of the increasing availability of genome-scale data. ANI finds shared genes between pairs of genomes using bidirectional BLAST+ and estimates the nucleotide-level similarity between these genomes by taking the sum of all nucleotide matches (i.e., percent identity multiplied by alignment length) and dividing this by the total length of all putative orthologues (Goris et al., 2007). We used the JSpecies 3.1.2 webserver (http://jspecies.ribohost.com/jspeciesws/#home; accessed March 2020) to apply ANI (Richter & Rosselló-Móra, 2009; Richter et al., 2016). We used pyani 0.2.10 to visualize this comparison (Pritchard et al., 2016). GGD also uses bidirectional BLAST+ to find high-scoring segment pairs (HSPs) between pairs of genomes. These HSPs are then converted to distances using one of three formulas (Meier-Kolthoff et al., 2014). We report the results of the recommended formula (*d*_4_) that divides the sum of identical nucleotides by HSP length (as opposed to genome length) to calculate distance for each putative ortholog. We used the GGDC 2.1 webserver (https://ggdc.dsmz.de/home.php; accessed March 2020) to apply GGD and its associated DNA-DNA hybridization value prediction (Meier-Kolthoff et al., 2014). We applied both methods to our assemblies of strains *P. agricolaris* BaQS159, *P. hayleyella* BhQS11, and *P. bonniea* BbQS859, and control *Paraburkholderia* chosen from the alignment-free tree: *P. fungorum* ATCC BAA-463, *P. fungorum* FDAARGOS245, and *P. insulsa* LMG28183 for *P. agricolaris* BaQS159, and *P. megapolitana* LMG23650 and *P. phenoliruptrix BR3459a* for *P. hayleyella* BhQS11 and *P. bonniea* BbQS859. All comparison taxa were chosen for their proximity to the type strains on our phylogeny (Fig. 1) and for their high quality draft genomes with relatively small numbers of assembled contigs. The latter two taxa are a compromise choice because no taxa within the subclade of *Paraburkholderia* containing *P. hayleyella* BhQS11 and *P. bonniea* BbQS859 (but not *P. eburnea* or *P. ferrariae*) are significantly more closely related to these two type strains on their own long branch than any other taxa (Fig. 1).

### Carbon Usage

We used Biolog GN2 Microplates to determine carbon source usage patterns for each bacterial strain (Biolog Inc., Hayward, CA). See Table 1 for strain names. These plates contain 95 test carbons and one blank control well. The 95 test carbons correspond to 11 carbon groups such as carbohydrates and amino acids (see Table S3 for complete list of carbon groups and individual carbons in each group). We brought the Biolog plates to room temperature prior to filling. We grew each bacterial isolate from frozen stocks on SM/5 agar plates for about 4 days to stationary phase at 22  °C. We collected a small amount of bacteria using a sterile loop and made suspensions in non-nutrient buffer (2.25 g KH_2_PO_4_ and 0.67 g K_2_HPO_4_ per liter DDH_2_O) for each bacterial isolate at an OD_600_ 0.7. We then added 150uL of this bacteria suspension to each well of the GN2 plate. Plates were incubated at 30 °C for 24 h, at which point they were photographed and the optical density was measured using a Tecan Infinite M200Pro plate reader (Wavelength = 590 nm, bandwidth = 9 nm, 5 flashes per well). We scored the results from the Biolog tests as follows –either the well was positive, meaning that the bacteria could use the substance as a carbon source, or the well was negative and it was not an available carbon source. To determine if a well was positive or negative, we calculated if the absorbance of each well minus the blank control absorbance is above or below 97.5% of the blank control. This is equivalent to a 5% confidence for a two-tailed distribution. We determined the negative baseline independently for each plate based on the value of the blank control. A summary of individual carbon source utilization can be found in Table S4.

**Table 1:** Summary table of comparisons for all species, including a subset of carbon usage types. We show the specific strain names for the three *Paraburkholderia* sp. nov. and the reference *Paraburkholderia* strains, the number of *Paraburkholderia* sp. nov. isolates in each group with the type strain in bold, whether or not the isolates are associated with *D. discoideum* (positive association indicated by a + sign), average bacterial length, optimal growth temperature, and metabolic tests. Next, a subset of about one-fourth of the relevant carbon types follows. *P. agricolaris* and *P. bonniea* are able to utilize some carbons that their close *Paraburkholderia* relatives cannot. A plus (+) symbol indicates all isolates were able to use a specific carbon and a minus (−) symbol indicates that they could not use that carbon. A number value indicates the percentage of strains in a specific group that can utilize that particular carbon. Specifics for each individual strain can be found in the supplemental information (raw data files).

Characteristic	*P. fungorum* ATCC BAA-463	*P. xenovorans* LB400	*P. phymatum* STM-815	*P. agricolaris* sp. nov	*P. hayleyella* sp. nov	*P.bonniea* sp. nov
n (# strains)	1	1	1	7	7	2
*Dictyostelium* symbionts	–	–	–	+	+	+
*Paraburkholderia* sp. nov. strain names; Type strains are in bold	Not applicable	Not applicable	Not applicable	BaQS31, BaQS70, **BaQS159**, BaQS175, BaQS317, BaQS983, BaQS1007	**BhQS11**, BhQS21, BhQS22, BhQS46, BhQS115, BhQS155, BhQS530	BbQS433, **BbQS859**
Cell length (µm)	1.57 ± 0.03	1.66 ± 0.03	1.7 ± 0.03	1.45 ± 0.01	1.36 ± 0.01	1.38 ± 0.01
Optimal growth temperature (°C)	37	30 and 37	37	30	30	30
Metabolic test: Catalase activity	+++	+++	+++	+	+++	+++
Metabolic test: Nitrate reduction	Positive: Nitrate to Nitrite	Postive: Nitrate to Nitrite to N_2_	Positive: Nitrate to Nitrite	Positive: Nitrate to Nitrite	Positive: Nitrate to Nitrite	Positive: Nitrate to Nitrite
Metabolic test: Oxidase activity	+	+	+	+	+	+
Maltose	–	–	–	43%	–	–
D-Cellobiose	–	–	–	71%	–	–
α-D-Lactose	–	–	–	71%	–	–
γ-Hydroxy Butyric Acid	–	+	–	57%	–	–
Inosine	–	–	–	86%	–	+
D-Melibiose	+	**–**	**–**	**–**	**–**	**–**
Xylitol	+	–	+	57%	–	–
Glycyl-L-Aspartic Acid	+	+	–	71%	–	–
Gentiobiose	+	+	–	57%	–	50%
Glucuronamide	+	+	+	57%	–	–
α-Keto Valeric Acid	+	+	+	71%	–	–
2-Aminoethanol	+	+	+	71%	–	–
D-Galacturonic Acid	+	+	+	86%	–	–
L-Fucose	+	+	+	+	29%	–
D-Galactose	+	+	+	+	–	50%
Mono-Methyl-Succinate	+	+	+	+	–	50%
N-Acetyl-D-galactosamine	+	+	+	+	–	–
L-Arabinose	+	+	+	+	–	–

We analyzed all data using R v3.4.1 (R Core Team, 2018) employing the following specific packages (Fox & Weisberg, 2011; Freeman & Halton, 1951). We performed a principal component analysis on the ability of individual members of each *Paraburkholderia* sp. nov. and three near sister reference *Paraburkholderia* to utilize carbon sources grouped into 11 carbon types (see Table S3 for individual carbons in each group). To test the effect of carbon usage by *Paraburkholderia* species, we used a generalized linear mixed model with a random-slope and a binomial error distribution. We used *Paraburkholderia* clone as our random factor, with clade and carbon type as fixed factors, and ability to use a particular carbon source as the response. We also compared each carbon source between species pairs (Table S5) with *post hoc* comparisons and Benjamini–Hochberg adjusted *P*-values *(Benjamini & Hochberg, 1995).*

### Fatty acid composition

We collected cells for fatty acid analysis by scraping cellular material from agar plates using a sterilized inoculation loop. The cellular material was collected in glass vials (which had been baked to remove organic contaminants) then lyophilized and weighed. Attempts were made to minimize the amount of SM5 agar scraped by the loop, but a media blank was processed via the same procedure to assess media contributions to fatty acids. Fatty acids were simultaneously extracted from samples and trans-esterified to fatty acid methyl esters (FAMEs) Rodriguez-Ruiz et al., 1998, using a mixture of hexane, methanol, and acetyl chloride heated to 100 °C for 10 min, followed by addition of more hexane to induce a phase separation, and remove the lipid phase. FAMEs were analyzed by gas chromatography-mass spectrometry using a HP 7890 gas chromatograph fitted with a split/splitless injector operated in splitless mode, equipped with a J&W DB-5 fused silica capillary column (30 m length, 0.25-mm inner diameter, and 0.25- µm film thickness) and coupled to an Agilent 6973 mass selective detector. Identification of individual FAMEs was based on mass spectra and retention times. The abundance of each fatty acid was determined by integrating its peak area relative to a co-injected standard (methyl tetracosanoate). Unsaturated fatty acids were further analyzed by converting FAMEs to their dimethyl disulfide adducts (Nichols, Guckert & White, 1986) and analyzing by the same method used to analyze the FAMEs.

### Bacterial length

To examine morphological characteristics, we grew each bacterial isolate from frozen stocks on SM/5 agar plates (Douglas et al., 2013) for about 4 days to stationary phase at 22 °C. We then collected and prepared a bacterial suspension of each test isolate in non-nutrient buffer at OD_600_1.5. To prepare fixed bacteria for imaging by microscopy, we first prepared the fixative solution by adding 6.26 µL of 8% gluteraldehyde per one mL of 16% paraformaldehyde (Electron Microscopy Sciences, Hatfield PA USA). Next, we added 200 µL of each bacterial suspension, 8 µL of 1M NaPO_4_pH 7.4, and 40 µL of the fixative solution to a 1.5 ml. Eppendorf tube and gently mixed. We incubated the reactions for 15 min at room temperature, followed immediately by 30 min on ice. We then centrifuged briefly at 10,000 g to pellet and wash the bacteria, repeating 3 times using phosphate buffered saline (PBS; Fisher Scientific, Pittsburg PA, USA), and ultimately resuspended in 1ml PBS. We prepared the microscope slides for image capture by adding 200 µL of 1% agarose in PBS (melted and slightly cooled) onto a single-depression microscope slide (VWR, Radnor PA, USA) and immediately overlaid with a cover slip. After 10 min of cooling, we removed the cover slip and added 5 µL of the fixed bacteria samples directly onto the agarose pad. Once the bacteria solution dried, the coverslip was replaced. We captured images and measured the lengths of about 100 individual bacteria for each isolate using a Nikon TI-E microscope and NIS-Elements software (see Table S6 for exact number measured for each isolate). Using R (Bates et al., 2015)*,* we compared bacterial lengths among each isolate using a generalized linear mixed model with a random-slope and a negative binomial error distribution. We used *Paraburkholderia* clone as our random factor and clade as fixed factor, with cell length as the response. We made *post hoc* comparisons to test the effect of differences in bacterial length between species pairs using Benjamini–Hochberg adjusted *P*-values (Benjamini & Hochberg, 1995).

### Metabolic tests

We used the three type strains and three reference *Paraburkholderia* (*P. fungorum ATCC BAA-463, P. xenovorans LB400, and P. phymatum STM-815*) to assess catalase activity, oxidase activity, and nitrate reduction using assay kits from Becton-Dickinson (Sparks, MD). The tests were performed on bacteria plated from frozen stock onto SM/5 agar plates and grown for about 48 h at 22 °C.

### Optimal growth temperature and growth range

We used all strains of *P. agricolaris*, P. *hayleyella*, *P. bonniea*, and three reference *Paraburkholderia* (*P. fungorum ATCC BAA-463, P. xenovorans LB400, and P. phymatum STM-815*) to determine optimal growth temperature and growth range. We streaked all bacterial clones onto SM/5 nutrient agar plates, and grew them at 5 different temperatures (4 °C, 22 °C, 30 °C, 37 °C, and 45 °C). We examined the plates and photographed them at 24 h. We scored plate growth using the following categories: no growth, little growth, moderate growth, and excellent growth. We recorded optimal growth temperature for each isolate based on the temperature at which the bacteria grew the most densely (see Table S7 for growth data and Fisher’s Exact Contingency Table). We performed a Fisher’s Exact Test with a 4 × 3 matrix testing the 22 °C, 30 °C, and 37 ° C temperature data to look for correlations between growth range and extent of growth among the four groups of *Paraburkholderia (Freeman & Halton, 1951).* We excluded 4 °C and 45  °C data from the analysis because none of the isolates including the controls grew at these temperatures.

### Antibiotic sensitivity tests

We tested the three *Paraburkholderia* sp. nov. type strains for antibiotic sensitivity using antibiotic sensitivity disk sets from Carolina Biological Supply Co. (Burlington, North Carolina). We prepared a bacterial suspension of each type strain in non-nutrient buffer at OD_600_ 1.5 and plated 200 µl onto a SM/5 agar test plate. We divided the plate into quadrants and placed one antibiotic test disk plus one blank disk in each of the four quadrants. We repeated the test twice and scored each antibiotic and blank disk for zones of inhibition after 48 h of growth at 22 °C. We measured and recorded the diameter of any inhibition zones present. See Table S8 for measurements and species descriptions for results.

## Results

### Phylogenomic analyses indicate three separate symbiont species

Draft assemblies of all three type genomes were resolved to contain two scaffolds. *P. agricolaris* BaQS159 is estimated to have a genome of approximately 8.7 megabase pairs (Mbp), which is similar size to other closely related *Paraburkholderia* (e.g., *P. fungorum* ATCC BAA-463 has a genome size of 9 Mbp) while *P. hayleyella* BhQS11 and *P. bonniea* BbQS859 are estimated to have a genome of less than half this size at approximately 4.1 Mbp each (Table S9). In addition to their smaller genome size, *P. hayleyella* and *P. bonniea* each have lower GC content (59.0 mol%) compared to *P. agricolaris* (62.0 mol%). Next, we used k-mer based alignment-free phylogeny reconstruction and two different *in silico* methods of genome comparison, Average Nucleotide Identity (ANI) and Genome-Genome Distance (GGD), to understand the phylogenetic placement of these *Burkholderia* and species delineation respectively. Both types of methods improve upon the shortfalls of species delineation using 16S rRNA only as they can take into account sequence characteristics of entire genomes (Janda & Abbott, 2007). We also generated a full-length 16S rRNA phylogeny for reference.

We estimated the phylogenetic relationships of the symbionts relative to other known plant-associated and environmental *Paraburkholderia*. The alignment-free phylogenetic analysis using frequencies of *k*-mers across the 72 *Burkholderia* sensu lato finished and draft genomes consistently supported the close relationship of *P. agricolaris* BaQS159 with *P. fungorum* ATCC BAA-463, *P. fungorum* FDAARGOS 245, and *P. insulsa* LMG28183, as well as the sister relationship between *P. hayleyella* BhQS11 and *P. bonniea* BbQS859 (Fig. 1). In addition, *P. hayleyella* BhQS11 and *P. bonniea* BbQS859 are on their own long branch. The topology of this tree, including genus-level relationships, is consistent with a recent phylogeny of *Burkholderia* sensu lato using 106 conserved proteins for 92 taxa (Buekes et al., 2017). Though not necessarily accurate (Felsenstein, 1985; Efron, Halloran & Holmes, 1996), nonparametric bootstraps indicated that our topology was robust, given the data as all bootstrap values were 64 or higher within the subclade of *Paraburkholderia* containing our three type strains (Fig. 1). The Type (Strain) Genome Server currently only contains 18 closely related *Paraburkholderia* type strains but confirmed that *P. fungorum* and *P. insulsa* are the most closely related type strains to *P. agricolaris* BaQS159, while *P. hayleyella* BhQS11 and *P. bonniea* BbQS859 are no more closely related to any other type strains than they are to *P. agricolaris* BaQS159 (Table S10).

ANI and GGD are both methods developed to improve upon traditional DNA-DNA hybridization (DDH) methods of delineating new prokaryotic species (Richter & Rosselló-Móra, 2009; Thompson et al., 2013). Conventional thresholds for species delineation are 95% for ANI and 70% for GGD-based predicted DDH values for consideration as the same species. *P. agricolaris* BaQS159 was quite similar in both ANI and predicted DDH to *P. fungorum* ATCC BAA-463 and *P. insulsa* LMG28183, but still at levels below thresholds for same species consideration (Tables 2A, 3A). Both *P. hayleyella* BhQS11 and *P. bonniea* BbQS859 were significantly divergent from *P. megapolitana* LMG23650 and *P. phenoliruptrix BR3459a* based on ANI and predicted DDH (Tables 2B, 3B). The Hadamard matrix representing ANI multiplied by alignment length also indicated that our type strains are significantly different from the comparison strains (Fig. S1).

**Table 2:** Average nucleotide identity (ANI) between pairs of the proposed type strains of *Paraburkholderia* sp. nov. isolates associated with *D. discoideum* and closely related *Paraburkholderia*. These ANI scores (and percent aligned nucleotide) indicate that (a) *P. agricolaris* is a separate species from *P. fungorum* and *P. insulsa*, and that (b) *P. hayleyella* and *P. bonniea* are separate from each other and from *P. megapolitana* and *P. phenoliruptrix*. The conventional ANI threshold for prokaryotic species delineation is 95%.

(a) ANIb	*P. agricolaris* BaQS159	*P. insulsa LMG28183*	*P. fungorum ATCC BAA-463*	*P. fungorum FDAARGOS245*
*P. agricolaris* BaQS159	–	94.04 (75.05)	94.02 (75.69)	94.01 (75.65)
*P. insulsa LMG28183*	93.86 (67.96)	–	97.73 (83.15)	97.37(81.90)
*P. fungorum ATCC BAA-463*	93.99 (72.70)	97.84 (88.11)	–	97.74 (87.42)
*P. fungorum FDAARGOS245*	94.09 (73.21)	97.72 (87.05)	97.99 (87.55)	–

(b) ANIb	*P. hayleyella* BhQS11	*P. bonniea BbQS859*	*P. megapolitana LMG23650*	*P. phenoliruptrix BR3459a*
*P. hayleyella* BhQS11	–	76.67 (58.09)	76.60 (44.98)	76.69 (44.70)
*P. bonniea BbQS859*	79.55 (59.74)	–	76.34 (46.97)	76.25 (46.18)
*P. megapolitana LMG23650*	75.30 (26.59)	75.19 (27.14)	–	78.11 (43.13)
*P. phenoliruptrix BR3459a*	75.49 (26.33)	75.34 (26.53)	78.28 (42.48)	–

**Table 3:** Predicted DNA-DNA hybridization (DDH) values based on Genome-to-Genome Distance (GGD) between proposed type strains of *Paraburkholderia* sp. nov. isolates associated with *D. discoideum* and closely related *Paraburkholderia*. These scores (and confidence intervals in brackets) indicate that (a) *P. agricolaris* is a separate species from *P. fungorum* and *P. insulsa*, and that (b) *P. hayleyella* and *P. bonniea* are separate from each other and from *P. megapolitana* and *P. phenoliruptrix*. The conventional DDH threshold for prokaryotic species delineation is 70%.

(a) GGD	*P. insulsa LMG28183*	*P. fungorum ATCC BAA-463*	*P. fungorum FDAARGOS245*
*P. agricolaris* BaQS159	65.1 [62.2–67.9]	64.8 [61.9–67.6]	64.6 [61.7–67.4]
*P. insulsa LMG28183*	–	87.9 [85.3–90]	85.8 [83.2–88.1]
*P. fungorum ATCC BAA-463*	–	–	87.0 [84.4–89.2]

(b) GGD	*P. bonniea BbQS859*	*P. megapolitana LMG23650*	*P. phenoliruptrix BR3459a*
*P. hayleyella* BhQS11	23.6 [21.3–26.1]	21.5 [19.2–23.9]	22.1 [19.9–24.6]
*P. bonniea BbQS859*	–	21.1 [18.8–23.5]	22.0 [19.7–24.5]
*P. megapolitana LMG23650*	–	–	23.7 [21.4–26.2]

The full-length 16S rRNA phylogeny confirms ours and community-wide concerns with relying on a single gene phylogeny to determine species relationships. The close relationship between *P. agricolaris* BaQS159 and *P. fungorum* ATCC BAA-463 and FDAARGOS245 and *P. insulsa* LMG28183 is recovered, as is the sister relationship between *P. hayleyella* BhQS11 and *P. bonniea* BbQS859 (Fig. S2). All intragenomic 16S copies clustered together within each symbiont genome as expected (Coenye & Vandamme, 2003; Acinas et al., 2004; Pei et al., 2010). However, the long branches of species such as *P. megapolitana* LMG23650, *P. rhizosphaerae* LMG29544, and *P. phenazium* LMG2247 (Fig. 1) show their effect in this single gene tree. As noted above, the alignment-free tree recovers the majority of species relationships from a recent tree of 92 species of *Burkholderia* sensu lato (Buekes et al., 2017). However, these long branch taxa are found in incongruous locations on the 16S tree, for example *P. megapolitana* LMG23650 and *P. phenazium* LMG2247 are sister taxa and found nested within the clade containing *P. fungorum* ATCC BAA-463 and FDAARGOS245 and *P. agricolaris* BaQS159 (Fig. S2). We are able to confirm with this tree that no *Paraburkholderia* species are natural controls for comparison to *P. hayleyella* BhQS11 and *P. bonniea* BbQS859.

### Carbon usage for *P. hayleyella* and *P. bonniea* is greatly reduced

**Figure 2:**
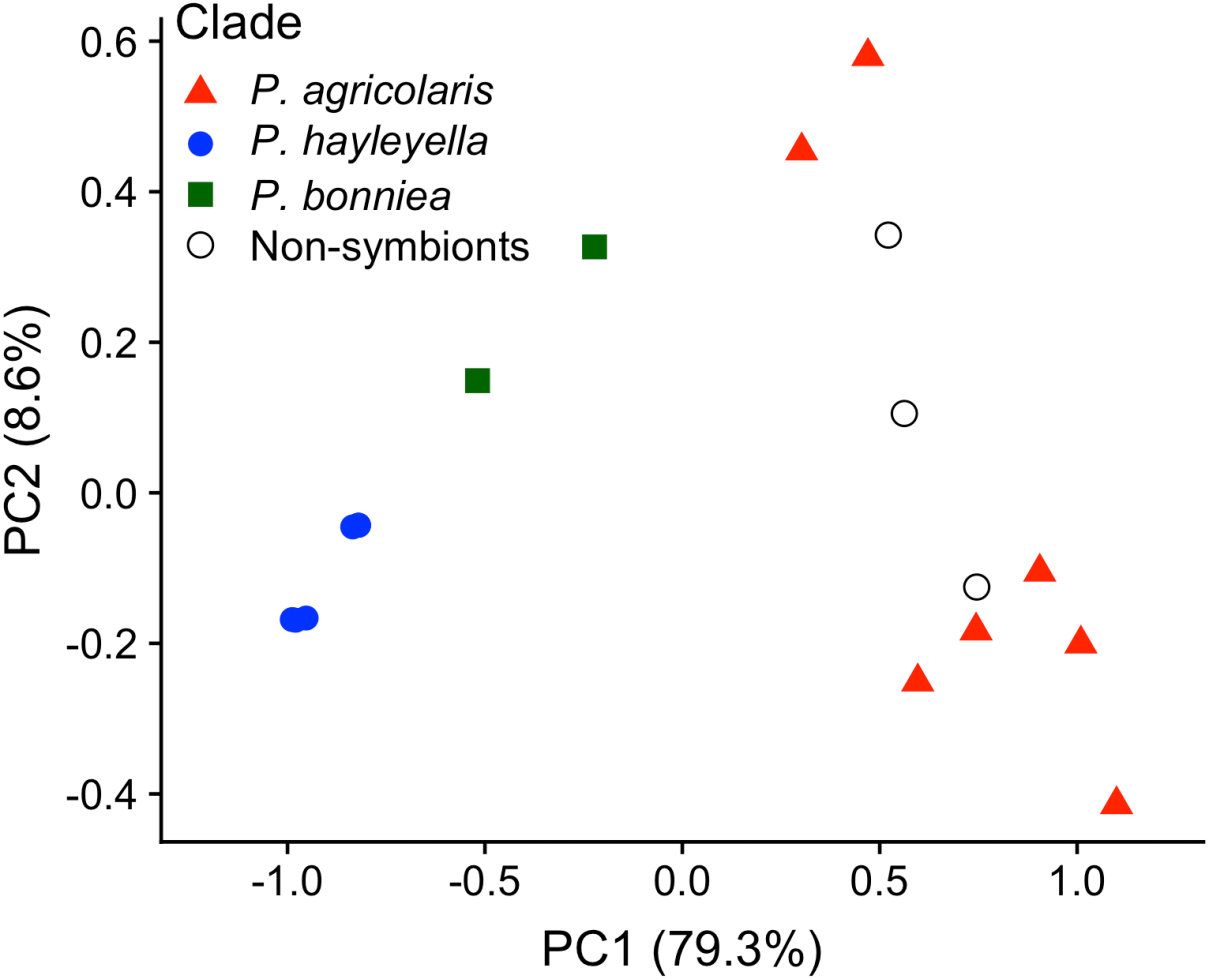
Carbon usage varies among *Paraburkholderia* symbionts with a pattern of loss in *P. hayleyella* and *P. bonniea*. Principal Component 1 (*x*-axis) accounts for 79.3% of the variance and Principal Component 2 (*y*-axis) accounts for 8.6% of the variance. Each symbol represents one bacteria isolate with different symbols representing each species. A higher value on the *x*-axis represents a larger number of carbon sources that can be utilized; *P. hayleyella* and *P. bonniea* have greatly reduced carbon usage compared to *P. agricolaris* and the non-symbionts.

We tested strains from the three *Paraburkholderia* sp.nov. for carbon usage employing Biolog GN2 test plates. See Table 1 for specific strains used. The 95 carbon types from these test plates are organized into eleven functional groups (Table S3). We subjected results from these functional groups to principal component analysis (PCA) to query for significant differences between the three *Paraburkholderia* sp. nov. (Fig. 2). The generalized output of the PCA revealed that most of the variation could be described by PC1 (79.3%) and PC2 (8.6%). PC1 is composed of ten of the eleven components and positively correlates roughly equally with nine of these components (carbohydrates, esters, carboxylic acids, amides, amino acids, aromatic chemicals, amines, alcohols, and phosphorylated chemicals) suggesting these nine criteria vary together, with a tendency towards loss in *P. hayleyella* and *P. bonniea*. PC2 is composed of six components and positively correlates strongly with two (phosphorylated chemicals and amines). One component of the PCA analysis, brominated chemicals, was utilized by all of the bacteria so was not useful in distinguishing between the carbon groups. The scatter plot of the component scores for PC1 and PC2 shows that the control *Paraburkholderia* species and *P. agricolaris* sp. nov. can use a broader range of carbon sources than *P. hayleyella* or *P. bonniea* (Fig. 2). We found the most variance in carbon use was explained by an interaction between *Paraburkholderia* species and carbon source type (*χ*^2^ = 497.43, DF = 43, *P* ≪ 0.001, ΔAIC = −175.0). We also found an overall effect of both additive terms in the model, *Paraburkholderia* clade (*χ*^2^ = 58.54, DF = 3, *P* ≪ 0.001, ΔAIC = −52.5) and carbon type (*χ*^2^ = 383.94, DF = 10, *P* ≪ 0.001, ΔAIC = −363.9), over the null model. From our model, we found *P. agricolaris*, *P. hayleyella*, and *P. bonniea* are all significantly different from one another (Benjamini–Hochberg adjusted *p*-values: *P. agricolaris*/ *P. hayleyella P* ≪ 0.001, *P. agricolaris*/ *P. bonniea P* ≪ 0.001, *P. hayleyella*/ *P. bonniea P* = 0.003). Specific differences can be found in the species descriptions.

We found *P. hayleyella* and *P. bonniea* are significantly different in overall carbon usage compared to three near sister control *Paraburkholderia* species while *P. agricolaris* are not (Benjamini–Hochberg adjusted *p*-values: *P. agricolaris p* = 0.34, *P. hayleyella P* ≪ 0.001, *P. bonniea P* ≪ 0.001). However, though carbon usage by *P. agricolaris* is not statistically different from the control *Paraburkholderia*, Table 1 and Table S4 highlight differences in usage of specific carbons by *P. agricolaris* sp. nov. These include no utilization of D-melibiose by *P. agricolaris* sp. nov. compared to *P. fungorum* ATCC BAA-463*,* and no utilization of adonitol by *P. agricolaris* sp. nov. compared to *P. xenovorans* LB400*.* Additionally, many or all of *P. agricolaris* sp. nov. are able to utilize several carbons sources that *P. fungorum* ATCC BAA-463 cannot. Examples are: 100% *P. agricolaris* sp. nov. tested can utilize hydroxy-L-proline, 86% tested can utilize L-ornithine and inosine, and 71% tested can utilize glycogen, D-cellobiose, and α-D-lactone (Table 1 and Table S4).

We also found differences in the overall pattern of carbon use when we performed pairwise comparisons between species for each of the individual carbon source types (Table S5). From these pairwise carbon use comparisons, some patterns emerge. The divergence in carbon use for *P. hayleyella* and *P. bonniea* from the reference *Paraburkholderia* species and *P. agricolaris* is predominantly in their use of amino acids, carbohydrates, and carboxylic acids. However, *P. hayleyella* and *P. bonniea* diverge from each other in their use of amides and carbohydrates. *P. agricolaris* and *P. hayleyella* also diverge in their use of amides. *P. agricolaris* has maintained a similar carbon use pattern as the reference outgroup species, but it appears to be diverging in its use of aromatic chemicals, specifically inosine.

### Fatty acid compositions for symbiont *Paraburkholderia* type strains contain large quantities of cyclopropane fatty acids (CPA)

Fatty acid results are shown in Table 4 and detailed in *Paraburkholderia* sp. nov. descriptions for exact proportions. Minor fatty acids (abundances <0.2%) are not reported. All three *Paraburkholderia* sp. nov. showed similar fatty acid patterns, dominated by three fatty acids, C16:0 and two cyclopropane fatty acids (CPA), 17:CPA and 19:CPA. We found much higher amounts proportionally of 17:CPA and 19:CPA in the three type strains than have been reported for our three non-symbiont control *Paraburkholderia*. There were minor quantitative differences among the strains, with BbQS859 containing more 16:0 and 17:CPA, but less 19:CPA than the other two strains.. The SM/5 growth media contained 16:1. The amount of media contaminating bacterial biomass could not be quantified, but is estimated to be much lower than 10% of the total mass. At 10% of the total mass, up to one-third of the 16:1 would have derived from the media. In addition to FAMEs, chromatograms of all three strains contained diploptene, suggesting the presence of hopanoids, but these were not further analyzed.

**Table 4:** Fatty acid composition of three *Paraburkholderia* sp. nov. The *Paraburkholderia* sp. nov. type strains contain large proportions of cyclopropane fatty acids (CPA) compared to non-symbiont control Paraburkholderia. The three symbiont Paraburkholderia type strains show similar fatty acid profiles. Minor fatty acids (abundances < 0.2%) are not reported.

**Fatty acid composition**						
	**Symbiont*Paraburkholderia*sp. nov.**			**Reference*Paraburkholderia***		
	*P. agricolaris* BaQS159	*P. hayleyella* BhQS11	*P. bonniea* BaQS859	*P. fungorum* (9 strains)^a^	*P. xenovorans* LB400^b^	*P. phymatum* STM815^c^
**Fatty acid**						
C14:0	0.8%	1.0%	1.0%	4.6 ± 0.1%	3.7%	4.6%
C14:0 3-OH					4.7%	8.7%
C16:1	0.4%*	0.7%*	0.5%*		20.0%	22.6%
C16:0	19.5%	22.1%	25.8%	14.7 ± 0.9%	18.0%	19.6%
C17:CPA	31.6%	32.4%	38.6%	5.1 ± 1.6%	2.3%	4.9%
C16:1 2-OH				3.5 ± 0.7%	1.5%	1.6%
C16:0 2-OH	0.2%	0.2%	0.2%	3.6 ± 0.5%	1.7%	1.0%
C16:0 3-OH				5.6 ± 0.5%	3.9%	6.6%
C18:1 *ω7*	3.2%	5.2%	2.1%	35.6 ± 2.1%	39.1%	28.5%
C18:0	0.7%	0.6%	1.6%		1.0%	
C19:CPA	43.5%	37.9%	30.1%	2.5 ± 0.7%	2.3%	1.2%
C18:1 2-OH				1.7 ± 0.2%	0.8%	<1.0%

**Notes.**

* SM/5 growth media contained C16:1. Media could have contributed up to one third of C16:1 at high estimates of media contamination of biomass collected for symbiont Paraburkholderia sp. nov.

References for nonsymbiont Paraburkholderia controls a Coenye et al. (2001). b Fain & Haddock (2001). c Vandamme et al. (2002).

### Catalase activity is reduced for *P. agricolaris* type strain

We tested the three *Paraburkholderia* sp. nov. type strains and three reference *Paraburkholderia* as controls to determine distinguishing metabolic markers. All six *Paraburkholderia* are positive for catalase activity. Interestingly, the type strain for *P. agricolaris* BaQS159 is weakly positive (+) for catalase compared to the strongly positive response (+++) of the other two *Paraburkholderia* sp. nov. as well as the three control *Paraburkholderia*. The ability to reduce nitrate to nitrite is present in all three of the *Paraburkholderia* sp. nov. type strains and *P. fungorum* ATCC BAA-364 and *P. phymatum* STM815. However, *P. xenovorans* LB400 is able to reduce nitrate to nitrite to nitrogen gas. Additionally, all six *Paraburkholderia* were positive for oxidase activity indicating cytochrome c oxidase is present.

### Symbiont bacteria length is shorter

**Figure 3:**
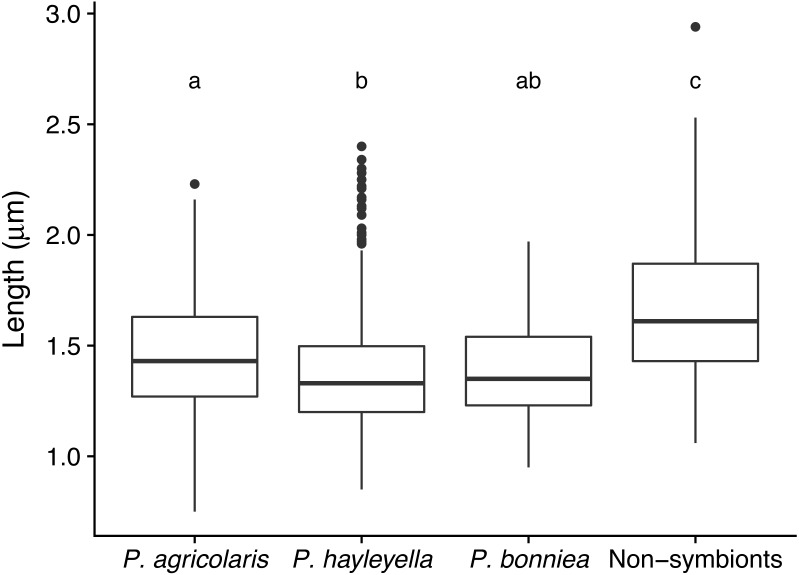
Symbiont *Paraburkholderia* bacteria lengths are shorter than non-symbionts. We measured the length of about one hundred bacteria for each *Paraburkholderia* sp. nov. and for the nonsymbionts (see Table S6). We used seven strains for *P. agricolaris*, seven strains for *P. hayleyella*, two strains for *P. bonniea*, and three strains for non-symbionts. On average, we found all three symbiont bacteria species are significantly shorter than non-symbiont bacteria species. Significant differences in length found between bacteria are indicated by different letters which reflect results of a Benjamini-Hochberg correction for multiple comparisons.

We examined morphological differences between the new *Paraburkholderia* species and reference *Paraburkholderia* controls by measuring bacterial length (Fig. 3 and Table S6). The generalized linear mixed model showed an overall difference in the effect of *Paraburkholderia* clade on length (*χ*^2^ = 17.19, DF = 3, *P* <0.001, ΔAIC = −11). Between species, we found that all *Paraburkholderia* sp. nov. are significantly shorter in length than the reference outgroup species (Benjamini–Hochberg adjusted *P-* values: *P. agricolaris P* = 0.0013, *P. hayleyella P* <<0.001, *P. bonniea P* = 0.0012). We also found that lengths differed between *P. agricolaris* and *P. hayleyella* (*P* = 0.033), but not between *P. agricolaris* and *P. bonniea* (*P* = 0.32) or between *P. hayleyella* and *P. bonniea* (*P* = 0.67).

### Optimal growth temperature for symbiont *Paraburkholderia* sp. nov. is reduced compared to near relative reference *Paraburkholderia*

**Figure 4:**
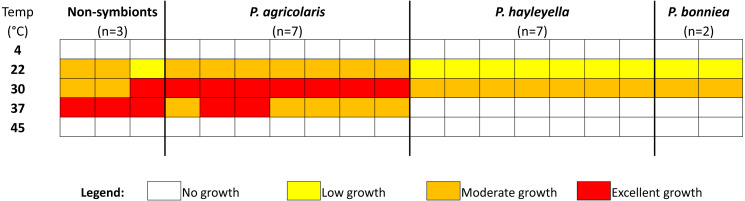
The optimal growth temperature of *Paraburkholderia* sp. nov. is 30 °C. We tested a range of temperatures to determine growth range and optimal temperature. *P. hayleyella* and *P. bonniea* have reduced range of growth and grow less densely compared to *P. agricolaris* and the non-symbionts. *P. agricolaris* has the same range as the three nonsymbiont *Paraburkholderia* but grows less densely at 37 °C and best at 30 °C compared to the non-symbionts.

We tested all isolates to determine the range of temperatures permissive for growth and the optimal growth temperature. We used a range of five temperatures: 4 °C, 22° C, 30 °C, 37 °C, and 45° C. For range of growth, we first found that none of our isolates including the reference *Paraburkholderia* controls were able to sustain visible growth at either 4 °C or 45 °C (Fig. 4). The optimal growth temperature for the three symbiont *Paraburkholderia* sp. nov. is 30 °C compared to the reference *Paraburkholderia* optimum of 37 °C. We found all isolates of *P. hayleyella* and *P. bonniea* grew less densely overall and had no growth at 37 °C compared to reference controls and *P. agricolaris*. *P. agricolaris* grew vigorously at 30 °C and moderately well at 37 °C compared to reference controls that had excellent growth at a higher optimal temperature of 37 °C. We performed a Fisher’s Exact Test comparing the 22 °C, 30 °C, and 37° C data and excluding the 4 °C and 45 °C because neither symbiont nor reference isolates grew at these two temperatures. The results of the exact contingency table test (Table S7) showed that the growth range and extent of growth differed among the four groups of *Paraburkholderia* (*P* < 0.001). We did post hoc tests to determine specific growth differences. Using a Bonferroni corrected cutoff of 0.008, *P. hayleyella* is significantly different from *P. agricolaris* (*P* = 0.000022) and the reference *Paraburkholderia* (*P* = 0.0032). *P. bonniea* is different from *P. agricolaris* (*P* = 0.0072), but not the controls (*P* = 0.063), or from *P. hayleyella* (*P* = 0.074). Lastly, *P. agricolaris* does not differ from the reference *Paraburkholderia* (*P* = 0.091).

### Descriptions of *Paraburkholderia agricolaris* sp. nov., *Paraburkholderiahayleyella* sp. nov., and *Paraburkholderia bonniea* sp. nov.

*Paraburkholderia agricolaris* (a’gri.co.la.ris. L. fem. adj. *agricolaris* facilitating farming). The morphology of colonies is off-white, domed, and shiny with smooth edges. Bacteria are motile, non-sporulating, straight rods. The G+C content varies between 61.5 and 61.9 mol% calculated from whole genomic sequences. The strains are stored in a sterile 20% glycerol solution at −80 °C and subcultured on SM/5 agar plates at 22 °C. We isolated the type strain BaQS159 as a symbiont of wild *D. discoideum* clone QS159 in May 2008. *D. discoideum* QS159 was isolated from soil and leaf litter collected from Mountain Lake Biological Station in April 2008. The G+C content of the type strain is 61.6 mol% calculated from the whole genomic sequence. Good growth at 30 °C, weak growth at 22 °C and 37 °C, and no growth at either 4 °C or 45 °C on nutrient agar containing glucose, bactopeptone, and yeast extract. The type strain BaQS159 is positive for oxidase activity, the ability to reduce nitrate to nitrite, and has slight catalase activity (+). BaQS159 contains the following fatty acids as percentage of total: C14:0 (0.8%), C16-2OH (0.2%), C16:1* (0.4%), C16:0 (19.5%), C17:CPA (31.6%), C18:1 *ω*7 (3.2%), C18:8 (0.7%), and C19:CPA (43.5%). *Growth media contained C16.1. At high estimates of media contamination of biomass, media could have contributed up to one third of C16.1. Additionally, the type strain BaQS159 has the ability to utilize the following carbon sources as determined by the Biolog GN2 test panel: Tween 40;Tween 80; N-Acetyl-D-galactosamine; N-Acetyl-D-glucosamine; Adonitol; L-Arabinose; D-Arabitol; D-Fructose; L-Fucose; D-Galactose; α-D-Glucose; m-Inositol; D-Mannitol; D-Mannose; L-Rhamnose; D-Sorbitol; Methyl Pyruvate; Mono-Methyl-Succinate; Acetic Acid; Cis-Aconitic Acid; Citric Acid; Formic Acid; D-Galactonic Acid Lactone; D-Gluconic Acid; D-Glucosaminic Acid; D-Glucuronic Acid; α-Hydroxy Butyric Acid; *β*-Hydroxy Butyric Acid; p-Hydroxy Phenylacetic Acid; α-Keto Butyric Acid; α-Keto Glutaric Acid; D,L-Lactic Acid; Malonic Acid; Propionic Acid; Quinic Acid; D-Saccharic Acid; Sebacic Acid; Succinic Acid; Bromo Succinic Acid; Succinamic Acid; L-Alaninamide; D-Alanine; L-Alanine; L-Alanyl-glycine; L-Asparagine; L-Aspartic Acid; L-Glutamic Acid; Glycyl-L-Glutamic Acid; L-Histidine; Hydroxy-L-Proline; L-Leucine; L-Ornithine; L-Phenylalanine; L-Proline; L-Pyroglutamic Acid; D-Serine; L-Serine; L-Threonine; γ-Amino Butyric Acid; Urocanic Acid; Inosine; Uridine; Glycerol. *Paraburkholderia agricolaris* BaQS159 is susceptible to tetracycline at 30 µg/ml, neomycin at 30 µg/ml, kanamycin at 30 µg/ml, and novobiocin at 30 µg/ml. BaQS159 is not susceptible to penicillin at 10 units, erythromycin at 30 µg/ml, streptomycin at 10 µg/ml and chloramphenicol at 30 µg/ml. The majority of characteristics for the type strain are in agreement with other six tested representatives of *P. agricolaris* sp. nov. We found differences in carbon source usage among the seven tested *P. agricolaris* sp. nov. (Table S4) and are as follows: dextrin (14%), glycogen (71%), D-cellobiose (71%), gentiobiose (57%), α-D-lactose (71%), lactulose (29%), maltose (43%), xylitol (57%), D-galactononic acid (86%), γ-hydroxy butyric acid (57%), itaconic acid (14C%), α-keto-valeric acid (71%), glucuronamide (14%), glycyl-L-aspartic acid (71%), L-ornithine (86%), D-L carnitine (71%), inosine (86%), phenyethylamine (29%), putrescine(29%), 2-aminoethanol (71%), D,L- α-glycerol phosphate (57%), and glucose-6-phosphate (71%).

Differences between *Paraburkholderia* sp.nov.:

Catalase activity is reduced (+) for *P. agricolaris* type strain compared to *P. hayleyella* BhQS11 (+++) and *P. bonniea* BbQS859 (+++).

*P. hayleyella* BhQS11 and *P. bonniea* BbQS859 are susceptible to erythromycin, streptomycin, and chloramphenicol but *P. agricolaris* type strain is not.

*P. agricolaris* BaQS159 is able to utilize N-Acetyl-D-galactosamine, N-Acetyl-D-glucosamine, Adonitol, L-Arabinose, D-Arabitol, D-Fructose, D-Galactose, m-Inositol, D-Mannitol, L-Rhamnose, D-Sorbitol, Mono-Methyl-Succinate, Cis-Aconitic Acid, Citric Acid, Formic Acid, D-Galactonic Acid Lactone, D-Glucosaminic Acid, D-Glucuronic Acid, p-Hydroxy Phenylacetic Acid, Malonic Acid, Quinic Acid, D-Saccharic Acid, Sebacic Acid, Succinamic Acid, L-Alaninamide, L-Alanine, L-Histidine, Hydroxy-L-Proline, L-Leucine, L-Ornithine, L-Phenylalanine, L-Pyroglutamic Acid, D-Serine, γ-Amino Butyric Acid, Urocanic Acid, Inosine, Uridine, and Glycerol which *P. hayleyella* BhQS11 cannot.

*P. agricolaris* BaQS159 is able to utilize N-Acetyl-D-galactosamine, Adonitol, L-Arabinose, D-Arabitol, L-Fucose, D-Galactose, m-Inositol, D-Mannitol, L-Rhamnose, D-Sorbitol, Mono-Methyl-Succinate, Acetic Acid, Formic Acid, D-Galactonic Acid Lactone, D-Glucuronic Acid, p-Hydroxy Phenylacetic Acid, Malonic Acid, Quinic Acid, D-Saccharic Acid, Sebacic Acid, L-Histidine, Hydroxy-L-Proline, L-Ornithine, L-Phenylalanine, L-Pyroglutamic Acid, D-Serine, γ-Amino Butyric Acid, Urocanic Acid, Uridine, and Glycerol which *P. bonniea* BbQS859 cannot.

*Paraburkholderia hayleyella* (hay’ley.el.la. N.L. fem. adj. *hayleyella*, pertaining to Hayley). Colony morphology is off-white, domed, and shiny with smooth edges. Bacteria are motile, non-sporulating, straight rods. The strains are stored in a sterile 20% glycerol solution at −80 °C and subcultured on SM/5 agar plates at 22 °C. We isolated the type strain BhQS11 as a symbiont of wild *D. discoideum* clone QS11 in February 2008. *D. discoideum* QS11 was isolated from soil and leaf litter collected from Mountain Lake Biological Station in October 2000. The G+C content is 59.24 mol% calculated from whole genomic sequence. Good growth at 30 °C, weak growth at 22 °C, and no growth at either 4 °C, 37 °C, or 45 °C on nutrient agar containing glucose, bactopeptone, and yeast extract. The type strain BhQS11 is positive for oxidase activity, the ability to reduce nitrate to nitrite, and has strong catalase activity (+++). BaQS11 contains the following fatty acids as percentage of total: C14:0 (1.0%), C16-2OH (0.2%), C16:1* (0.7%), C16:0 (22.1%), C17:CPA (32.4%), C18:1 *ω*7 (5.2%), C18:8 (0.6%), and C19:CPA (37.9%). *Growth media contained C16.1. At high estimates of media contamination of biomass, media could have contributed up to one third of C16.1. Additionally, the type strain BhQS11 has the ability to utilize the following carbon sources as determined by the Biolog GN2 test panel: Tween 40; Tween 80; L-Fucose; α-D-Glucose; D-Mannose; Methyl Pyruvate; Acetic Acid; D-Gluconic Acid; α-Hydroxy Butyric Acid; *β*-Hydroxy Butyric Acid; α-Keto Butyric Acid; α-Keto Glutaric Acid; D,L-Lactic Acid; Propionic Acid; Succinic Acid; Bromo Succinic Acid; D-Alanine; L-Alanyl-glycine; L-Asparagine; L-Aspartic Acid; L-Glutamic Acid; Glycyl-L-Glutamic Acid; L-Proline; L-Serine; L-Threonine. *Paraburkholderia hayleyella* BhQS11 is susceptible to tetracycline at 30 µg/ml, neomycin at 30 µg/ml, kanamycin at 30 µg/ml, erythromycin at 30 µg/ml, novobiocin at 30µg/ml, streptomycin at 10 µg/ml, and chloramphenicol at 30 µg/ml. BhQS11 is not susceptible to penicillin at 10 units. The majority of characteristics for the type strain are in agreement with other six tested representatives of *P. hayleyella* sp. nov. The slight differences in carbon source usage among the seven tested *P. hayleyella* sp. nov. (Table S4) we found are as follows: D-fructose (14%), L-fucose (29%), D-mannose (86%), D-glucosaminic acid (57%), succinamic acid (14%), L- alaninamide (43%), D-alanine (43%), L-alanine (71%), D-serine (43%), L-threonine (86%), and γ-amino butyric acid (29%).

Differences between *Paraburkholderia* sp.nov.: Catalase activity is strong (+++) for *P. hayleyella* BhQS11 compared to *P. agricolaris* BaQS159 (+). BhQS11 is susceptible to erythromycin, streptomycin, and chloramphenicol but *P. agricolaris* type strain is not. *P. hayleyella* BhQS11 is able to utilize L-Fucose and Acetic Acid which *P. bonniea* BbQS859 cannot. *P. hayleyella* BhQS11 utilizes a smaller subset of the same carbons as *P. agricolaris* BaQS159.

*Paraburkholderia bonniea* (bonn’-ie-a. N.L. fem. adj. *bonniea*, pertaining to Bonnie). Colonies are off-white, shiny, and domed with smooth edges. Bacteria are motile, non-sporulating, straight rods. The strains are stored in a sterile 20% glycerol solution at −80 °C and subcultured on SM/5 agar plates at 22 °C. We isolated the type strain BbQS859 as a symbiont of wild *D. discoideum* clone QS859 in August 2014. *D. discoideum* QS859 was isolated from deer feces collected from Mountain Lake Biological Station in July 2014. The G+C content of the type strain is 58.7 mol% calculated from whole genomic sequence. Good growth at 30 °C, weak growth at 22 °C, and no growth at either 4 °C, 37 °C, or 45 °C on nutrient agar containing glucose, bactopeptone, and yeast extract. The type strain BbQS859 is positive for oxidase activity, the ability to reduce nitrate to nitrite, and has strong catalase activity (+++). BbQS859 contains the following fatty acids as percentage of total: C14:0 (1.0%), C16-2OH (0.2%), C16:1* (0.5%), C16:0 (25.8%), C17:CPA (38.6%), C18:1 *ω*7 (2.1%), C18:8 (1.6%), and C19:CPA (30.1%). *Growth media contained C16.1. At high estimates of media contamination of biomass, media could have contributed up to one third of C16.1. Additionally, the type strain BbQS859 has the ability to utilize the following carbon sources as determined by the Biolog GN2 test panel: Tween 40; Tween 80; N-Acetyl-D-glucosamine; D-Fructose; α-D-Glucose; D-Mannose; Methyl Pyruvate; Mono-Acetic Acid; Cis-Aconitic Acid; Citric Acid; D-Gluconic Acid; D-Glucosaminic Acid; α-Hydroxy Butyric Acid; *β*-Hydroxy Butyric Acid; α-Keto Butyric Acid; α-Keto Glutaric Acid; D,L-Lactic Acid; Propionic Acid; Succinic Acid; Bromo Succinic Acid; Succinamic Acid; L-Alaninamide; D-Alanine; L-Alanine; L-Alanyl-glycine; L-Asparagine; L-Aspartic Acid; L-Glutamic Acid; Glycyl-L-Glutamic Acid; L-Leucine; L-Proline; L-Serine; L-Threonine; Inosine. *Paraburkholderia bonniea* BbQS859 is susceptible to tetracycline at 30 µg/ml, neomycin at 30 µg/ml, kanamycin at 30 µg/ml, erythromycin at 30 µg/ml, novobiocin at 30 µg/ml, streptomycin at 10 µg/ml, chloramphenicol at 30 µg/ml. BbQS859 is not susceptible to penicillin at 10 units. The majority of characteristics for the type strain are in agreement with the one other representative of *P. bonniea* sp. nov. We found a few differences in carbon source usage and they are as follows: Type strain BbQS859 is able to utilize α-keto glutaric acid while strain BbQS433 cannot. BbQS433 is able to utilize D-galactose, gentiobiose, mono-methyl-succinate, L-histidine, D- serine, γ-amino butyric acid, and urocanic acid while type strain BbQS859 cannot.

Differences in carbon utilization between *Paraburkholderia* sp.nov.: Catalase activity is strong (+++) for *P. bonniea* BbQS859 compared to *P. agricolaris* BaQS159 (+). BbQS859 is susceptible to erythromycin, streptomycin, and chloramphenicol but *P. agricolaris* type strain is not. *P. bonniea* BbQS859 is able to utilize N-Acetyl-D-glucosamine, D-Fructose, Mono-Acetic Acid, Cis-Aconitic Acid, Citric Acid, Succinamic Acid, L-Alaninamide, L-Alanine, L-Leucine, and Inosine which *P. hayleyella* BhQS11 cannot. *P. bonniea* BbQS859 is able to utilize Mono-Acetic Acid which *P. agricolaris* BaQS159 cannot.

## Discussion

We use multiple lines of evidence to delineate *P. agricolaris*, *P. hayleyella*, and *P. bonniea* as new species. We had previously tested close 16S relatives and found only these three sp. nov. have the ability to colonize *D. discoideum*, to be carried through multiple amoebae-to-fruiting body cycles, and to facilitate the carriage of food bacteria to seed new environments (Brock et al., 2011; DiSalvo et al., 2015; Shu et al., 2018a; Shu et al., 2018b; Haselkorn et al., 2019). Now, using whole genome data, we place these three new species in a phylogeny (Fig. 1), which confirms previous phylogenetic analyses of 16S and multilocus sequence typing results (DiSalvo et al., 2015; Haselkorn et al., 2019). Pairwise genome comparisons of the three type strains to other closely related *Paraburkholderia* species indicate significant genomic divergence in ANI and GGD that warrant consideration as separate species (Tables 2 and 3). In further support, we analyzed several physical and metabolic traits and found significant differences between symbiont and non-symbiont *Paraburkholderia* (Table 1)*.* These data support the identification and naming of these *D. discoideum*-associated symbionts as three new *Paraburkholderia* species.

Our three *Paraburkholderia* sp. nov. have a facultative endosymbiotic lifestyle with their host *D. discoideum*. A common feature of endosymbiosis is the streamlining and loss of non-essential genes (Moran, McCutcheon & Nakabachi, 2008). Several lines of evidence suggest cell size corresponds positively with genome size. Examples are found in red blood cells and genome size of vertebrates where the red blood cell increases with genome size (Gregory, 2001). Using avian genomes known to be small and streamlined compared to other vertebrates, Organ et al. (2007) found a similar pattern of correspondence between fossilized osteocytes and predicted genome size in extant vertebrates*.* Beaulieu et al. (2008) also found a similar pattern in a broad array of 101 angiosperms showing cell size and genome size scale positively, something that has proven generally true in plants (Šímová & Herben, 2012). Here, we detail the reduced genome sizes and demonstrate that the lengths of the symbiotic *Paraburkholderia* sp. nov. bacteria are smaller than their free-living close relatives (Fig. 3, Table S9). Moreover, the type strains of *P. hayleyella*, *P. bonniea,* and *P. agricolaris* have lost the ability to utilize many of the 95 carbons tested compared to the non-symbiont *Paraburkholderia* tested suggesting corresponding gene losses based on loss of function (Table S4). The three *Paraburkholderia* sp. nov. have also diverged from each other and non-symbionts in carbon usage (Fig. 2). Both *P. agricolaris* and *B bonniea* are able to utilize some carbons that non-symbiont *Paraburkholderia* cannot (Tables S4 & S5). These data taken together suggest genome streamlining of non-essential genes and potential adaptation to an intracellular environment for the three *Paraburkholderia* sp. nov. and are consistent with an endosymbiotic lifestyle.

Notably, the fatty acid profiles in these strains of *Paraburkholderia* had uncommonly large quantities of CPA (cyclopropane fatty acids) (Table 4). CPA were not reported in any of the 13 strains of *Burkholderia* analyzed by Galbraith et al. (1999). Small to moderate proportions of CPA (up to 15% of total fatty acid pool) were detected in five strains of *Burkholderia* analyzed by Coenye et al. (2001), five strains by Vandamme et al. (2002), and one strain analyzed by Goris et al. (2004). These results suggest that the large proportions of CPA found in these sp. nov. strains are unusual. In *Escherichia coli* and other bacteria, increased CPA synthesis occurs during the transition to growth at stationary phase (Cronan, 1968) and the *cfa* gene responsible for producing them is at least partially induced by the general stress response sigma factor, RpoS (Chang & Cronan, 1999). CPA synthesis is also induced under a wide variety of stress conditions such as high temperature, low oxygen tension, lower pH, and low oxygen tension (Knivett & Cullen, 1965). High proportions of CPA have been implicated in resistance to pH (Chang & Cronan, 1999; Kim et al., 2005) and other stresses (Muñoz Rojas et al., 2006). In these strains of *Paraburkholderia*, the unusually high amounts of CPA under our culture conditions may represent an adaptation to life a symbiont. Alternatively, the presence of abundant CPA may suggest that laboratory culture conditions induce a stress response in these strains.

Lastly, the optimal growth temperature of the three *Paraburkholderia* sp. nov. at 30 °C is lower than the reference *Paraburkholderia* control species at 37 °C (Fig. 4). One possible explanation could be that this is an adaptation to the endosymbiotic lifestyle. *D. discoideum* have a much lower optimal growth temperature range of 20−25 °C (Sussman, 1956).

## Conclusions

In sum, these three new species have diverged from their ancestors in measurable ways that are likely to be due to their endosymbiotic habit within *D. discoideum* (Table 1)*.* We classify these isolates as novel species for which the names *Paraburkholderia agricolaris*, *Paraburkholderia hayleyella*, and *Paraburkholderia bonniea* are proposed with the strains BaQS159 (Dictybase DBS0351125; NCTC 14075), BhQS11 (Dictybase DBS0351126; NCTC 14077), and BbQS859 (Dictybase DBS0351127; NCTC 14076) respectively, designated as the type strains.

##  Supplemental Information

10.7717/peerj.9151/supp-1 Supplemental Information 1 Supplemental Figures and Tables

10.7717/peerj.9151/supp-2 Supplemental Information 2 Optimal Bacteria Growth Data

10.7717/peerj.9151/supp-3 Supplemental Information 3 Raw Data Carbon Usage

10.7717/peerj.9151/supp-4 Supplemental Information 4 Individual Values for Bacterium Length

## Additional Information and Declarations

### DNA Deposition

The following information was supplied regarding the deposition of DNA sequences: Draft genome sequences for type strains Paraburkholderia agricolaris BaQS159, Paraburkholderia hayleyella BhQS11, and Paraburkholderia bonniea BbQS859 are available at NCBI under BioProject PRJNA480875: QPEQ00000000, QPER00000000, QPES00000000. The raw PacBio sequencing reads are available under the same BioProject: SRR9134255–SRR9134257. The proposed type strains for Paraburkholderia agricolaris, Paraburkholderia hayleyella, and Paraburkholderia bonniea are available at Dictybase and NCTC: BaQS159: DBS0351125; NCTC 14075; BhQS11: DBS0351126; NCTC 14077; and BbQS859: DBS0351127; NCTC 14076.

### Data Availability

The following information was supplied regarding data availability: The raw data files are available in the Supplemental Files. The sequences are available at NCBI: SRR9134255–SRR9134257.

### New Species Registration

The following information was supplied regarding the registration of a newly described species: The proposed type strains for Burkholderia agricolaris, Burkholderia hayleyella, and Burkholderia bonniea are available at Dictybase and NCTC: BaQS159: DBS0351125, NCTC 14075; BhQS11: DBS0351126, NCTC 14077; and BbQS859: DBS0351127, NCTC 14076.
http://www.dictybase.org/db/cgi-bin/dictyBase/phenotype/strain_and_phenotype_details.pl?genotype_id=10845

http://www.dictybase.org/db/cgi-bin/dictyBase/phenotype/strain_and_phenotype_details.pl?genotype_id=10844

http://www.dictybase.org/db/cgi-bin/dictyBase/phenotype/strain_and_phenotype_details.pl?genotype_id=10846.
