# Supplementary material for: Endosymbiotic adaptations in three new bacterial species associated with Dictyostelium discoideum: Paraburkholderia agricolaris sp. nov., Paraburkholderia hayleyella sp. nov., and Paraburkholderia bonniea sp. nov
Source: PeerJ. 2020 May 22;8:e9151. doi: 10.7717/peerj.9151 (PMC7247526; doi:10.7717/peerj.9151)
Supplement: Supplemental Information 1 [file peerj-08-9151-s001.docx]

Endosymbiotic adaptations in three new bacterial species associated with *Dictyostelium discoideum*: *Paraburkholderia agricolaris* sp. nov., *Paraburkholderia hayleyella* sp. nov., and *Paraburkholderia bonniea* sp. nov.

Debra A. Brock^1*+^, Suegene Noh^2*^, Alicia N.M. Hubert^1^, Tamara S. Haselkorn^3^, Susanne DiSalvo^4^, Melanie K. Suess^5^, Alexander S. Bradley^5,6^, Mahboubeh Tavakoli-Nezhad^1^, Katherine S. Geist^1^, David C. Queller^1^, Joan E. Strassmann^1^

^1^Department of Biology, Washington University in St. Louis, Saint Louis, MO USA

^2^Department of Biology, Colby College, Waterville, ME USA

^3^Department of Biology, University of Central Arkansas, Conway, AK USA

^4^Department of Biology, Southern Illinois University Edwardsville, Edwardsville, IL USA

^5^Department of Earth and Planetary Sciences, Washington University in St. Louis, Saint Louis MO USA

^6^Division of Biology and Biomedical Sciences, Washington University in St. Louis, Saint Louis MO 63130

* These authors contributed equally to this work.

**^+^**Corresponding author

**Supplementary Files**

**Figure S1**

**
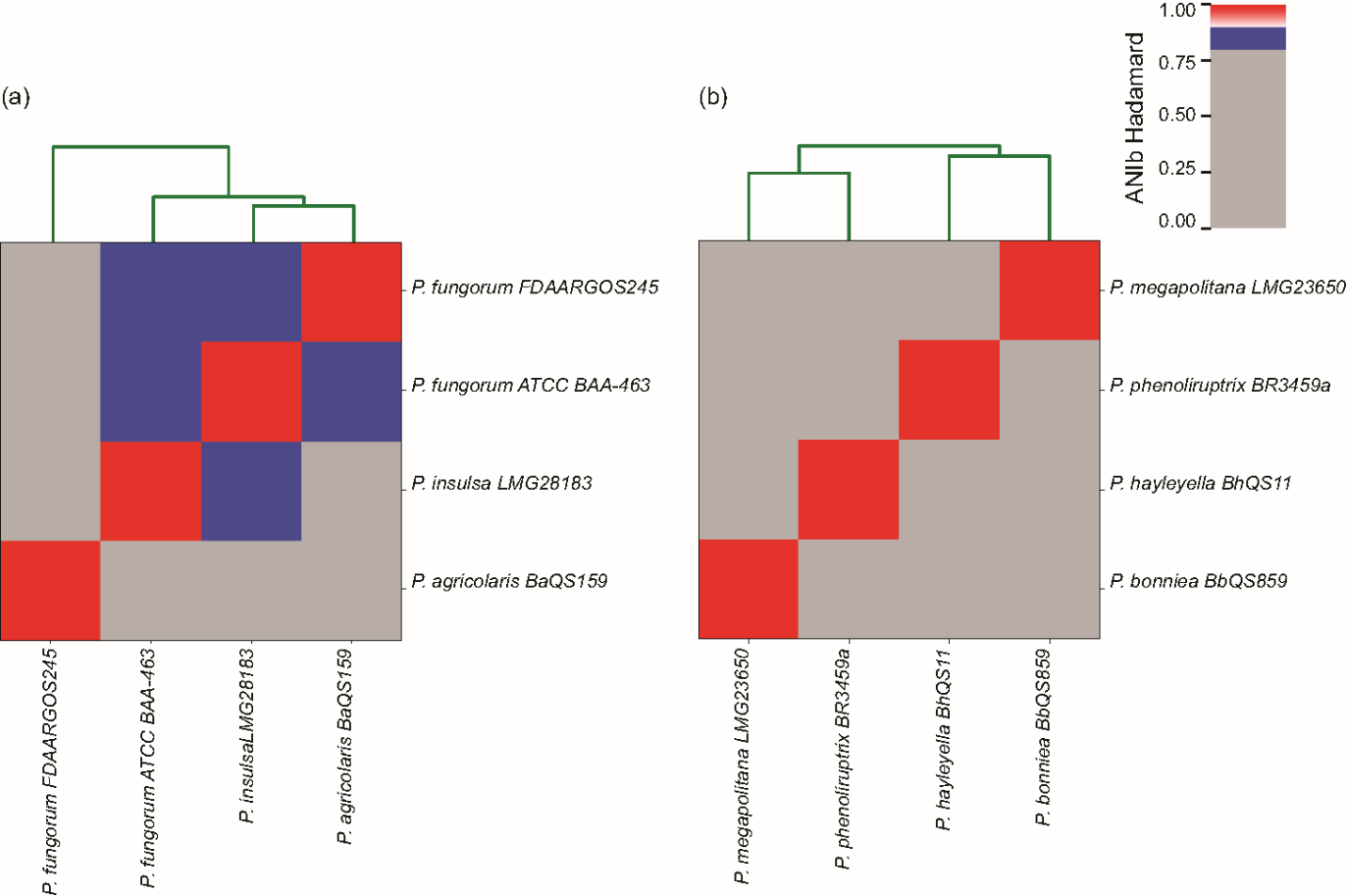
**

**Figure S1.** The Hadamard matrix representing ANI multiplied by alignment length between proposed type strains of *Paraburkholderia* sp. nov. isolated associated with *D. discoideum* and closely related *Paraburkholderia*. These scores indicate that our type strains are significantly different from the comparison strains. (a) *P. agricolaris* is a separate species from *P. fungorum* and *P. insulsa*. (b) *P. hayleyella* and *P. bonniea* are separate from each other and from *P. megapolitana* and *P. phenoliruptrix*.

**Figure S2**

**

**

**Figure S2.** Full-length 16S ribosomal RNA phylogeny including three new non-pathogenic *Paraburkholderia* species found in association with *D. discoideum* and *Burkholderia* sensu lato species. We aligned all 16S copies found in these new species with high quality 16S copies of type strains of *Burkholderia* sensu lato species, with a focus on the genera *Paraburkholderia* and *Caballeronia*. *Ralstonia pickettii* was used as the outgroup. All sequences other than those for the new species were downloaded from the SILVA database and aligned in a rRNA secondary structure aware manner. This tree is an approximately-Maximum Likelihood tree based on this alignment.

**Table S1**

| ***D. discoideum* clone** | **Bacteria name/species** | **Location** | **GPS Coordinates** | ***Paraburkholderia* sp. nov,**  **Type Strain** |
| --- | --- | --- | --- | --- |
| QS31 | BaQS31  *P. agricolaris* | Texas- Houston Arboretum | N 29° 46’, W 95° 27’ |  |
| QS70 | BaQS70  *P. agricolaris* | Texas- Houston Arboretum | N 29° 46’, W 95° 27’ |  |
| QS159 | BaQS159  *P. agricolaris* | Virginia- Mountain Lake Biological Station | N 37° 21’, W 80° 31’ | BaQS159 |
| QS175 | BaQS175  *P. agricolaris* | Texas- Houston Arboretum | N 29° 46’, W 95° 27’ |  |
| QS317 | BaQS317  *P. agricolaris* | Texas- Houston Arboretum | N 29° 46’, W 95° 27’ |  |
| QS983 | BaQS983  *P. agricolaris* | Virginia- Mountain Lake Biological Station | N 37° 21’, W 80° 31’ |  |
| QS1007 | BaQS1007  *P. agricolaris* | Virginia- Mountain Lake Biological Station | N 37° 21’, W 80° 31’ |  |
| QS11 | BhQS11  *P. hayleyella* | Virginia- Mountain Lake Biological Station | N 37° 21’, W 80° 31’ | BhQS11 |
| QS21 | BhQS21  *P. hayleyella* | Virginia- Mountain Lake Biological Station | N 37° 21’, W 80° 31’ |  |
| QS22 | BhQS22  *P. hayleyella* | Virginia- Mountain Lake Biological Station | N 37° 21’, W 80° 31’ |  |
| QS46 | BhQS46  *P. hayleyella* | Arkansas- Forest City | N 34˚ 50.1’, W 91˚28.1’ |  |
| QS115 | BhQS115  *P. hayleyella* | Indiana- Bloomington (Lobelia) | N 39° 13.2’, W 86° 20.5’ |  |
| QS155 | BhQS155  *P. hayleyella* | Virginia- Mountain Lake Biological Station | N 37° 21’, W 80° 31’ |  |
| QS530 | BhQS530  *P. hayleyella* | Indiana- Bloomington (Lobelia) | N 39° 13.2’, W 86° 20.5’ |  |
| QS433 | BbQS433  *P. bonniea* | Virginia- Mountain Lake Biological Station | N 37° 21’, W 80° 31’ |  |
| QS859 | BbQS859  *P. bonniea* | Virginia- Mountain Lake Biological Station | N 37° 37.6’, W 80° 51.9’ | BbQS859 |

**Table S1.** List of *Dictyostelium discoideum* clones, bacteria name and species identity isolated from each *D. discoideum* farmer, location collected along with GPS coordinates, and *Paraburkholderia* sp. nov. type strains. We used all *Paraburkholderia* sp. nov strains in experimental assays to determine carbon source utilization, *D. discoideum* spore length, and optimal temperature. Raw data for specific isolates can be found in supplementary data files. We used *Paraburkholderia* sp. nov. type strains in experimental assays to determine fatty acid composition, catalase activity, nitrate reduction, oxidase activity, and antibiotic sensitivity.

**Table S2**

| Genome name | NCBI Genomes | JGI Integrated Microbial Genomes |
| --- | --- | --- |
| *B. mallei* NCTC10247 |  | x |
| *B. pseudomallei* ATCC15682 |  | x |
| *B. pseudomallei* ATCC23343 |  | x |
| *C. choica LMG22940* | x |  |
| *C. concitans LMG29315* | x |  |
| *C. cordobensis LMG27620* | x |  |
| *C. glathei DSM50014* | x |  |
| *C. grimmiae R27* | x |  |
| *C. humi LMG22934* | x |  |
| *C. jiangsuensis MP1* | x |  |
| *C. megalochromosomata JC2949* | x |  |
| *C. sordidicola S170* | x |  |
| *C. telluris LMG22936* | x |  |
| *C. terrestris LMG22937* | x |  |
| *C. udeis LMG27134* | x |  |
| *C. zhejiangensis OP1* | x |  |
| *M. endofungorum* HKI465 |  | x |
| *M. rhizoxinica* HKI454 | x |  |
| *P. acidipaludis* NBRC101816 | x |  |
| *P. aromaticivorans* BN5 |  | x |
| *P. aspalathi* LMG27731 |  | x |
| *P. bannensis* NBRC103871 | x |  |
| *P. bryophila* 376MFSha3_1 | x |  |
| *P. caballeronis* LMG26416 |  | x |
| *P. caledonica* NBRC102488 | x |  |
| *P. caribensis* MWAP64 | x |  |
| *P. diazotrophica* LMG26031 |  | x |
| *P. dilworthii* WSM3556 | x |  |
| *P. dipogonis* ICMP19430 | x |  |
| *P. eburnea* LMG29537 |  | x |
| *P. ferrariae* NBRC106233 | x |  |
| *P. fungorum* ATCC BAA-463 | x |  |
| *P. fungorum* FDAARGOS245 |  | x |
| *P. ginsengisoli* NBRC100965 | x |  |
| *P. ginsengiterrae* DCY85 |  | x |
| *P. graminis* C4D1M | x |  |
| *P. heleia* NBRC101817 | x |  |
| *P. hospita* LMG20598 |  | x |
| *P. insulsa* LMG28183 |  | x |
| *P. kirstenboschensis* KB15 |  | x |
| *P. kururiensis thiooxydans* NBRC107107 | x |  |
| *P. megapolitana* LMG23650 |  | x |
| *P. mimosarum* LMG23256 | x |  |
| *P. monticola* JC2948 |  | x |
| *P. nodosa* DSM21604 | x |  |
| *P. oxyphila* NBRC105797 | x |  |
| *B. phenazium* LMG2247 |  | x |
| *P. phenoliruptrix* BR3459a | x |  |
| *P. phymatum* STM815 | x |  |
| *P. phytofirmans* PsJn | x |  |
| *P. piptadeniae* STM7183 |  | x |
| *P. rhizosphaerae* LMG29544 |  | x |
| *P. ribeironis* STM7296 |  | x |
| *P. sacchari* LMG19450 |  | x |
| *P. sartisoli* LMG24000 |  | x |
| *P. sediminicola* LMG24238 |  | x |
| *P. silvatlantica* SRCL-319 | x |  |
| *P. sprentiae* WSM5005 | x |  |
| *P. susongensis* LMG29540 |  | x |
| *P. terrae* NBRC100964 |  | x |
| *P. terricola* LMG20594 |  | x |
| *P. tropica* LMG22274 |  | x |
| *P. tuberum* DU833 | x |  |
| *P. unamae* MTI-641 | x |  |
| *P. xenovorans* LB400 | x |  |
| *R. pickettii* 12J | x |  |
| *T. caryophylli* Ballard720 |  | x |
| *T. soli* GP25-8 | x |  |
| *T. symbiotica* JPY-345 |  | x |

**Table S2.** Sources of 70 additional *Paraburkholderia* genomes and draft assemblies downloaded for phylogenomic analyses.

**Table S3.**

**
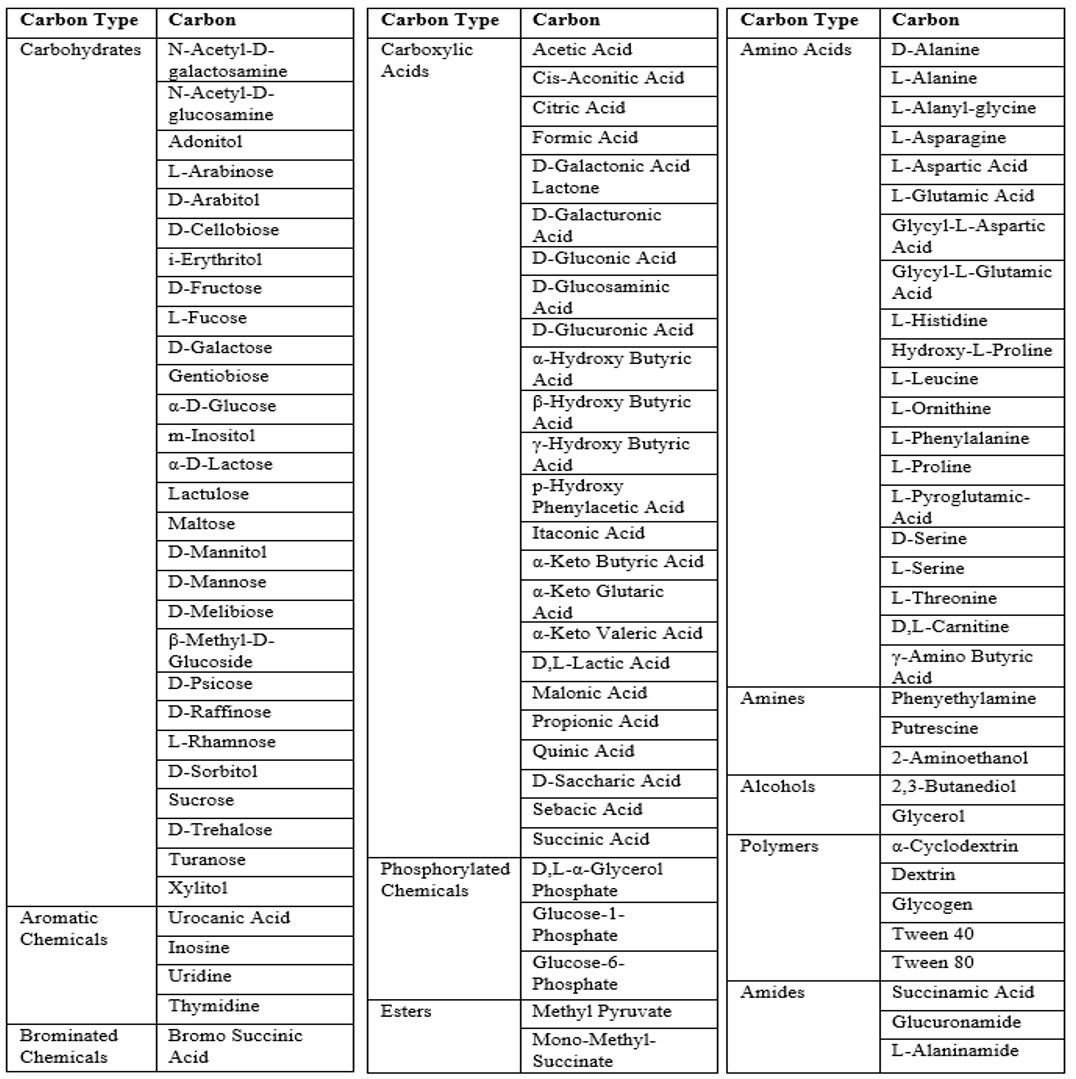
**

**Table S3.** Table of 95 test carbons from Biolog GN2 Microplates (Biolog Inc., Hayward, CA) arranged into groups of similar types.

**Table S4**

| **Characteristic** | | ***P. fungorum*** | ***P. xenovorans*** | ***P. phymatum*** | ***P. agricolaris***  **sp. nov** | ***P. hayleyella* sp. nov** | ***P. bonniea* sp. nov** |
| --- | --- | --- | --- | --- | --- | --- | --- |
| **n (# strains)** | | 1 | 1 | 1 | 7 | 7 | 2 |
| **Lab specific strain IDs** | | N/A | N/A | N/A | BaQS31, BaQS1007 BaQS70 BaQS175 BaQS159 BaQS317 BaQS983 | BhQS11 BhQS21 BhQS22 BhQS46 BhQS115BhQS155BhQS530 | BbQS433 BbQS859 |
| **Utilization of:** | |  |  |  |  |  |  |
| Polymers | α-Cyclodextrin | **-** | **-** | **-** | **-** | **-** | **-** |
|  | Dextrin | **-** | **-** | **-** | **14%** | **-** | **-** |
|  | Glycogen | **-** | **+** | **-** | **71%** | **-** | **-** |
|  | Tween 40 | **+** | **+** | **+** | **+** | **+** | **+** |
|  | Tween 80 | **+** | **+** | **+** | **+** | **+** | **+** |
| Carbohydrates | N-Acetyl-D-galactosamine | **+** | **+** | **+** | **+** | **-** | **-** |
|  | N-Acetyl-D-glucosamine | **+** | **+** | **+** | **+** | **-** | **+** |
|  | Adonitol | **+** | **-** | **+** | **+** | **-** | **-** |
|  | L-Arabinose | **+** | **+** | **+** | **+** | **-** | **-** |
|  | D-Arabitol | **+** | **+** | **+** | **+** | **-** | **-** |
|  | D-Cellobiose | **-** | **-** | **-** | **71%** | **-** | **-** |
|  | i-Erythritol | **-** | **-** | **-** | **-** | **-** | **-** |
|  | D-Fructose | **+** | **+** | **+** | **+** | **14%** | **+** |
|  | L-Fucose | **+** | **+** | **+** | **+** | **29%** | **-** |
|  | D-Galactose | **+** | **+** | **+** | **+** | **-** | **50%** |
|  | Gentiobiose | **+** | **+** | **-** | **57%** | **-** | **50%** |
|  | α-D-Glucose | **+** | **+** | **+** | **+** | **+** | **+** |
|  | m-Inositol | **+** | **+** | **+** | **+** | **-** | **-** |
|  | α-D-Lactose | **-** | **-** | **-** | **71%** | **-** | **-** |
|  | Lactulose | **-** | **-** | **-** | **29%** | **-** | **-** |
|  | Maltose | **-** | **-** | **-** | **43%** | **-** | **-** |
|  | D-Mannitol | **+** | **+** | **+** | **+** | **-** | **-** |
|  | D-Mannose | **+** | **+** | **+** | **+** | **86%** | **+** |
|  | D-Melibiose | **+** | **-** | **-** | **-** | **-** | **-** |
|  | β-Methyl-D-Glucoside | **-** | **-** | **-** | **-** | **-** | **-** |
|  | D-Psicose | **-** | **-** | **-** | **-** | **-** | **-** |
|  | D-Raffinose | **-** | **-** | **+** | **-** | **-** | **-** |
|  | L-Rhamnose | **+** | **+** | **+** | **+** | **-** | **-** |
|  | D-Sorbitol | **+** | **+** | **+** | **+** | **-** | **-** |
|  | Sucrose | **-** | **-** | **+** | **-** | **-** | **-** |
|  | D-Trehalose | **-** | **-** | **+** | **-** | **-** | **-** |
|  | Turanose | **-** | **-** | **-** | **-** | **-** | **-** |
|  | Xylitol | **+** | **-** | **+** | **57%** | **-** | **-** |
| Esters | Methyl Pyruvate | **+** | **+** | **+** | **+** | **+** | **+** |
|  | Mono-Methyl-Succinate | **+** | **+** | **+** | **+** | **-** | **50%** |
| Carboxylic Acids | Acetic Acid | **+** | **+** | **+** | **+** | **+** | **+** |
|  | Cis-Aconitic Acid | **+** | **+** | **+** | **+** | **-** | **+** |
|  | Citric Acid | **+** | **+** | **+** | **+** | **-** | **+** |
|  | Formic Acid | **+** | **+** | **+** | **+** | **-** | **-** |
|  | D-Galactonic Acid Lactone | **+** | **+** | **+** | **+** | **-** | **-** |
|  | D-Galacturonic Acid | **+** | **+** | **+** | **86%** | **-** | **-** |
|  | D-Gluconic Acid | **+** | **+** | **+** | **+** | **+** | **+** |
|  | D-Glucosaminic Acid | **+** | **+** | **+** | **+** | **57%** | **+** |
|  | D-Glucuronic Acid | **+** | **+** | **+** | **+** | **-** | **-** |
|  | α-Hydroxy Butyric Acid | **+** | **+** | **+** | **+** | **+** | **+** |
|  | β-Hydroxy Butyric Acid | **+** | **+** | **+** | **+** | **+** | **+** |
|  | γ-Hydroxy Butyric Acid | **-** | **+** | **-** | **57%** | **-** | **-** |
|  | p-Hydroxy Phenylacetic Acid | **+** | **+** | **+** | **+** | **-** | **-** |
|  | Itaconic Acid | **-** | **+** | **-** | **14%** | **-** | **-** |
|  | α-Keto Butyric Acid | **+** | **+** | **+** | **+** | **+** | **+** |
|  | α-Keto Glutaric Acid | **+** | **+** | **+** | **+** | **+** | **50%** |
|  | α-Keto Valeric Acid | **+** | **+** | **+** | **71%** | **-** | **-** |
|  | D,L-Lactic Acid | **+** | **+** | **+** | **+** | **+** | **+** |
|  | Malonic Acid | **+** | **+** | **+** | **+** | **-** | **-** |
|  | Propionic Acid | **+** | **+** | **+** | **+** | **+** | **+** |
|  | Quinic Acid | **+** | **+** | **+** | **+** | **-** | **-** |
|  | D-Saccharic Acid | **+** | **+** | **+** | **+** | **-** | **-** |
|  | Sebacic Acid | **+** | **+** | **+** | **+** | **-** | **-** |
|  | Succinic Acid | **+** | **+** | **+** | **+** | **+** | **+** |
| Brominated Chemicals | Bromo Succinic Acid | **+** | **+** | **+** | **+** | **+** | **+** |
| Amides | Succinamic Acid | **+** | **+** | **+** | **+** | **14%** | **+** |
|  | Glucuronamide | **+** | **+** | **+** | **57%** | **-** | **-** |
|  | L-Alaninamide | **+** | **+** | **+** | **+** | **43%** | **+** |
| Amino Acids | D-Alanine | **+** | **+** | **+** | **+** | **43%** | **+** |
|  | L-Alanine | **+** | **+** | **+** | **+** | **71%** | **+** |
|  | L-Alanyl-glycine | **+** | **+** | **+** | **+** | **+** | **+** |
|  | L-Asparagine | **+** | **+** | **+** | **+** | **+** | **+** |
|  | L-Aspartic Acid | **+** | **+** | **+** | **+** | **+** | **+** |
|  | L-Glutamic Acid | **+** | **+** | **+** | **+** | **+** | **+** |
|  | Glycyl-L-Aspartic Acid | **+** | **+** | **-** | **71%** | **-** | **-** |
|  | Glycyl-L-Glutamic Acid | **+** | **+** | **+** | **+** | **+** | **+** |
|  | L-Histidine | **+** | **+** | **+** | **+** | **-** | **50%** |
|  | Hydroxy-L-Proline | **-** | **+** | **+** | **+** | **-** | **-** |
|  | L-Leucine | **+** | **+** | **+** | **+** | **-** | **+** |
|  | L-Ornithine | **-** | **+** | **+** | **86%** | **-** | **-** |
|  | L-Phenylalanine | **+** | **+** | **+** | **+** | **-** | **-** |
|  | L-Proline | **+** | **+** | **+** | **+** | **+** | **+** |
|  | L-Pyroglutamic Acid | **+** | **+** | **+** | **+** | **-** | **-** |
|  | D-Serine | **+** | **+** | **+** | **+** | **43%** | **50%** |
|  | L-Serine | **+** | **+** | **+** | **+** | **+** | **+** |
|  | L-Threonine | **+** | **+** | **+** | **+** | **86%** | **+** |
|  | D,L-Carnitine | **+** | **+** | **+** | **71%** | **-** | **-** |
|  | γ-Amino Butyric Acid | **+** | **+** | **+** | **+** | **29%** | **50%** |
| Aromatic Chemicals | Urocanic Acid | **+** | **+** | **+** | **+** | **-** | **50%** |
|  | Inosine | **-** | **-** | **-** | **86%** | **-** | **+** |
|  | Uridine | **+** | **+** | **-** | **+** | **-** | **-** |
|  | Thymidine | **-** | **-** | **-** | **-** | **-** | **-** |
| Amines | Phenyethylamine | **-** | **-** | **+** | **29%** | **-** | **-** |
|  | Putrescine | **-** | **-** | **-** | **29%** | **-** | **-** |
|  | 2-Aminoethanol | **+** | **+** | **+** | **71%** | **-** | **-** |
| Alcohols | 2,3-Butanediol | **-** | **-** | **-** | **-** | **-** | **-** |
|  | Glycerol | **+** | **+** | **+** | **+** | **-** | **-** |
| Phosphorylated Chemicals | D,L-α-Glycerol Phosphate | **-** | **+** | **-** | **57%** | **-** | **-** |
|  | Glucose-1-Phosphate | **-** | **-** | **-** | **-** | **-** | **-** |
|  | Glucose-6-Phosphate | **-** | **+** | **-** | **71%** | **-** | **-** |

**Table S4.** Summary table of carbon source utilization for all species grouped by carbon type. A plus symbol indicates all isolates were able to use a specific carbon type and a minus symbol indicates no utilization. A number value indicates the percentage of isolates in a specific group that can utilize that particular carbon type. *P. agricolaris* and *P. bonniea* are able to utilize some carbon sources that their close *Paraburkholderia* relatives cannot. Specific information for all *Paraburkholderia* sp. nov. isolates can be found in the raw data files in the supplementary information.

**Table S5**

|  | **Pairwise Species Comparisons** | | | | | | |
| --- | --- | --- | --- | --- | --- | --- | --- |
|  |  | ***P. agricolaris* vs. Non-symbionts** | ***P. hayleyella* vs. Non-symbionts** | ***P. bonniea* vs. Non-symbionts** | ***P. agricolaris* vs. *P. hayleyella*** | ***P. agricolaris* vs. *P. bonniea*** | ***P. hayleyella* vs. *P. bonniea*** |
| **Carbon Source** | **Alcohols** |  |  |  |  |  |  |
|  | **Amides** |  |  |  | **0.0001** |  | **0.0381** |
|  | **Amines** |  |  |  |  |  |  |
|  | **Amino Acids** |  | **< 0.001** | **0.0003** | **< 0.001** | **< 0.001** |  |
|  | **Aromatic Chemicals** | **0.0817** |  |  | **0.0285** | **0.0899** |  |
|  | **Brominated Chemicals** |  |  |  |  |  |  |
|  | **Carbohydrates** |  | **< 0.001** | **< 0.001** | **< 0.001** | **< 0.001** | **0.0401** |
|  | **Carboxylic Acids** |  | **< 0.001** | **< 0.001** | **< 0.001** | **< 0.001** |  |
|  | **Esters** |  |  |  |  |  |  |
|  | **Phosphorylated Chemicals** |  |  |  |  |  |  |
|  | **Polymers** |  |  |  |  |  |  |
|  |  |  |  |  |  |  |  |
|  |  | **> 0.10** | **Levels of Significant Difference Between Carbon Use for *Paraburkholderia* outgroup species** | |  |  |  |
|  | . | **< 0.10** |  |  |  |  |  |
|  | * | **< 0.05** |  |  |  |  |  |
|  | ** | **< 0.01** |  |  |  |  |  |
|  | *** | **< 0.001** |  |  |  |  |  |

**Table S5**. Pairwise species comparisons of *Paraburkholderia* sp. nov. with each other and with three control outgroup *Paraburkholderia*. *P. hayleyella* and *P. bonniea* have diverged in carbon usage for amino acids, carbohydrates, and carboxylic acids compared to the outgroup species. The three *Paraburkholderia* sp. nov. have diverged from each other in pairwise carbon usage.

**Table S6**

| *Paraburkholderia* | Isolate | Number of bacteria measured |
| --- | --- | --- |
| *P. agricolaris* | BaQS31 | 110 |
|  | BaQS1007 | 126 |
|  | BaQS70 | 103 |
|  | BaQS175 | 114 |
|  | BaQS159 | 112 |
|  | BaQS317 | 128 |
|  | BaQS983 | 104 |
|  |  |  |
| *P. hayleyella* | BhQS11 | 95 |
|  | BhQS21 | 104 |
|  | BhQS22 | 105 |
|  | BhQS46 | 154 |
|  | BhQS115 | 125 |
|  | BhQS155 | 104 |
|  | BhQS530 | 111 |
|  |  |  |
| *P. bonniea* | BbQS433 | 110 |
|  | BbQS859 | 133 |
|  |  |  |
| Controls | *P. phymatum* | 104 |
|  | *P. fungorum* | 100 |
|  | *P. xenovorans* | 102 |

**Table S6.** Summary table of bacteria isolates used for length measurements and number measured for each isolate. Raw data for specific isolates can be found in supplementary information.

**Table S7**


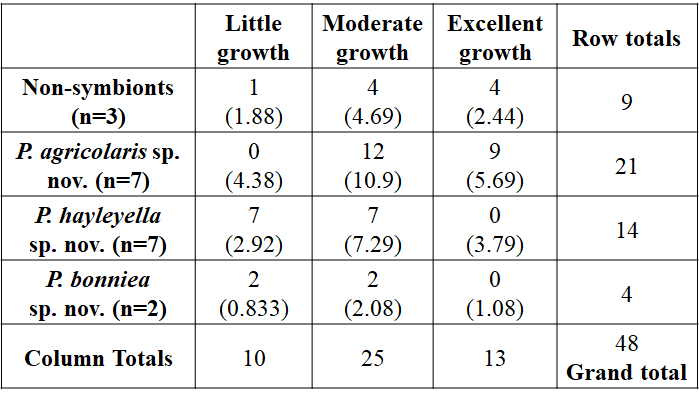


**Table S7.**  Fisher’s Exact Contingency Table. Each cell of this contingency table provides the following information for the growth data: the observed cell total for each growth category, and the expected cell total for growth category is below in parentheses. The given table has a probability of 1.1 E-07; the sum of the probabilities finds p < 0.001. Our hypothesis that growth rates are the same for all four groups is false.

**Table S8**


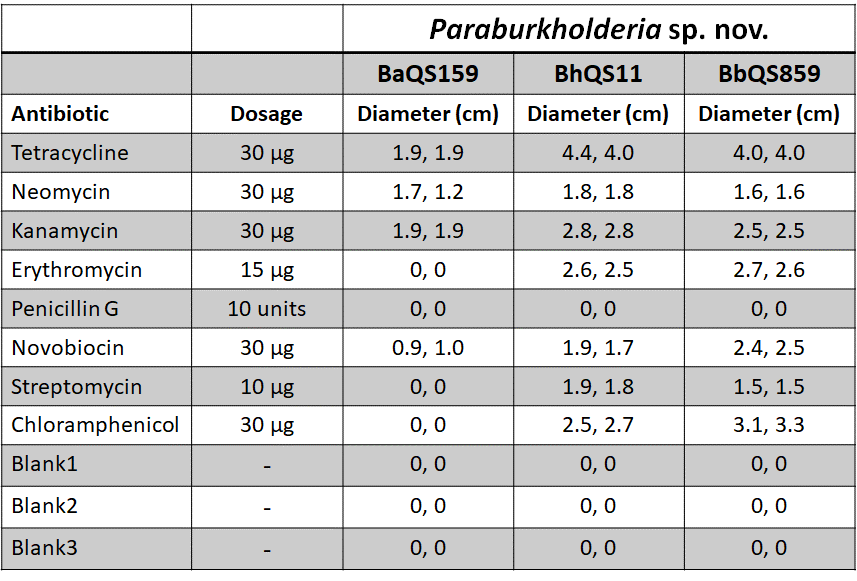


**Table S8. Antibiotic susceptibility test.** *Paraburkholderia* sp. nov. type strains were tested against eight antibiotics for susceptibility. We plated the bacteria on nutrient agar plates and measured the diameter of any zones of inhibition after 48 hours of bacterial growth at 22°C. Antibiotic and blank disk diameters equal 0.7cm.

**Table S9.**

|  | **Estimated genome size** | **Scaffold count** | **GC content** | **N50 contig length** | **Mean sequencing coverage** |
| --- | --- | --- | --- | --- | --- |
| *P. agricolaris* (BaQS159) | 8,762,141 | 2 | 0.62 | 4,840,882 | 122 |
| *P. hayleyella* (BhQS11) | 4,165,087 | 2 | 0.59 | 3,312,599 | 243 |
| *P. bonniea* (BbQS859) | 4,131,982 | 2 | 0.59 | 3,195,827 | 162 |

**Table S9.** Summary genome characteristics of draft genome assemblies for the three type strains. PacBio sequencing and assembly resulted in the resolution of these genomes into two scaffolds each.

**Table S10.**

| Type Strain Genome Server | | | | |
| --- | --- | --- | --- | --- |
| Query strain | **Subject strain** | **dDDH (%)** | **C.I. (%)** | **∆GC (%)** |
| BaQS159 | *Paraburkholderia insulsa* LMG 28183 | 65.1 | [62.2 - 67.9] | 0.02 |
| BaQS159 | *Paraburkholderia fungorum* NBRC 102489 | 64.8 | [61.9 - 67.7] | 0.23 |
| BaQS159 | *Paraburkholderia aspalathi* LMG 27731 | 37 | [34.5 - 39.5] | 0.52 |
| BaQS159 | *Paraburkholderia xenovorans* LB400 | 30.1 | [27.7 - 32.6] | 1.02 |
| BaQS159 | *Paraburkholderia phytofirmans* PsJN | 30 | [27.6 - 32.5] | 0.68 |
| BaQS159 | *Paraburkholderia aromaticivorans* BN5 | 30 | [27.6 - 32.5] | 1.33 |
| BaQS159 | *Paraburkholderia dipogonis* ICMP 19430 | 29.9 | [27.5 - 32.4] | 0.04 |
| BaQS159 | *Paraburkholderia ribeironis* STM 7296 | 29.2 | [26.8 - 31.7] | 0.87 |
| BaQS159 | *Paraburkholderia panaciterrae* DCY85-1 | 29 | [26.6 - 31.5] | 0.75 |
| BaQS159 | *Paraburkholderia ginsengiterrae* DCY85 | 29 | [26.6 - 31.5] | 0.89 |
| BaQS159 | *Paraburkholderia rhynchosiae* WSM3937 | 28 | [25.6 - 30.5] | 0.1 |
| BaQS159 | *Paraburkholderia sartisoli* LMG 24000 | 24.8 | [22.4 - 27.2] | 1.91 |
| BaQS159 | *Paraburkholderia caribensis* MWAP64 | 23.7 | [21.4 - 26.2] | 0.96 |
| BbQS859 | **BhQS11** | 23.6 | [21.3 - 26.1] | 0.65 |
| BaQS159 | *Paraburkholderia terrae* NBRC 100964 | 23.5 | [21.2 - 26.0] | 0.35 |
| BaQS159 | *Paraburkholderia megapolitana* LMG 23650 | 23.5 | [21.2 - 26.0] | 0.46 |
| BaQS159 | *Caballeronia telluris* LMG 22936 | 22.9 | [20.6 - 25.3] | 2.41 |
| BaQS159 | *Caballeronia udeis* LMG 27134 | 22.5 | [20.2 - 24.9] | 1.57 |
| BhQS11 | *Paraburkholderia dipogonis* ICMP 19430 | 22.1 | [19.8 - 24.5] | 2.27 |
| BhQS11 | *Paraburkholderia ribeironis* STM 7296 | 21.9 | [19.7 - 24.4] | 3.18 |
| BbQS859 | **BaQS159** | 21.9 | [19.6 - 24.3] | 2.96 |
| BhQS11 | *Paraburkholderia fungorum* NBRC 102489 | 21.9 | [19.6 - 24.3] | 2.54 |
| BbQS859 | *Paraburkholderia aromaticivorans* BN5 | 21.9 | [19.7 - 24.4] | 4.29 |
| BhQS11 | *Paraburkholderia phytofirmans* PsJN | 21.8 | [19.5 - 24.2] | 2.99 |
| BaQS159 | **BhQS11** | 21.8 | [19.5 - 24.2] | 2.31 |
| BhQS11 | *Paraburkholderia insulsa* LMG 28183 | 21.8 | [19.6 - 24.3] | 2.33 |
| BhQS11 | *Paraburkholderia xenovorans* LB400 | 21.8 | [19.6 - 24.3] | 3.33 |
| BaQS159 | *Caballeronia sordidicola* LMG 22029 | 21.8 | [19.6 - 24.3] | 1.46 |
| BbQS859 | *Paraburkholderia xenovorans* LB400 | 21.8 | [19.6 - 24.3] | 3.98 |
| BhQS11 | *Paraburkholderia aromaticivorans* BN5 | 21.7 | [19.5 - 24.2] | 3.64 |
| BhQS11 | *Paraburkholderia panaciterrae* DCY85-1 | 21.7 | [19.5 - 24.2] | 3.06 |
| BhQS11 | *Paraburkholderia ginsengiterrae* DCY85 | 21.7 | [19.5 - 24.2] | 3.2 |
| BbQS859 | *Paraburkholderia ginsengiterrae* DCY85 | 21.6 | [19.4 - 24.0] | 3.85 |
| BbQS859 | *Paraburkholderia panaciterrae* DCY85-1 | 21.6 | [19.4 - 24.1] | 3.71 |
| BbQS859 | *Paraburkholderia phytofirmans* PsJN | 21.6 | [19.4 - 24.0] | 3.64 |
| BhQS11 | *Paraburkholderia sartisoli* LMG 24000 | 21.6 | [19.4 - 24.1] | 4.23 |
| BbQS859 | *Paraburkholderia dipogonis* ICMP 19430 | 21.6 | [19.3 - 24.0] | 2.92 |
| BbQS859 | *Paraburkholderia ribeironis* STM 7296 | 21.5 | [19.3 - 24.0] | 3.83 |
| BhQS11 | *Paraburkholderia aspalathi* LMG 27731 | 21.5 | [19.3 - 24.0] | 1.8 |
| BbQS859 | *Paraburkholderia aspalathi* LMG 27731 | 21.5 | [19.3 - 23.9] | 2.45 |
| BbQS859 | *Paraburkholderia rhynchosiae* WSM3937 | 21.5 | [19.2 - 23.9] | 3.07 |
| BhQS11 | *Paraburkholderia megapolitana* LMG 23650 | 21.5 | [19.2 - 23.9] | 2.77 |
| BbQS859 | *Paraburkholderia insulsa* LMG 28183 | 21.4 | [19.1 - 23.8] | 2.98 |
| BhQS11 | *Paraburkholderia rhynchosiae* WSM3937 | 21.4 | [19.1 - 23.8] | 2.42 |
| BbQS859 | *Paraburkholderia fungorum* NBRC 102489 | 21.4 | [19.2 - 23.9] | 3.19 |
| BhQS11 | *Paraburkholderia caribensis* MWAP64 | 21.4 | [19.2 - 23.9] | 3.28 |
| BhQS11 | *Caballeronia telluris* LMG 22936 | 21.4 | [19.1 - 23.8] | 4.72 |
| BhQS11 | *Paraburkholderia terrae* NBRC 100964 | 21.3 | [19.0 - 23.7] | 2.66 |
| BbQS859 | *Paraburkholderia sartisoli* LMG 24000 | 21.2 | [19.0 - 23.7] | 4.88 |
| BbQS859 | *Paraburkholderia caribensis* MWAP64 | 21.2 | [19.0 - 23.7] | 3.93 |
| BbQS859 | *Caballeronia telluris* LMG 22936 | 21.1 | [18.8 - 23.5] | 5.37 |
| BbQS859 | *Paraburkholderia megapolitana* LMG 23650 | 21.1 | [18.8 - 23.5] | 3.42 |
| BbQS859 | *Paraburkholderia terrae* NBRC 100964 | 20.9 | [18.7 - 23.4] | 3.31 |
| BbQS859 | *Caballeronia sordidicola* LMG 22029 | 20.7 | [18.4 - 23.1] | 1.5 |
| BbQS859 | *Caballeronia udeis* LMG 27134 | 20.7 | [18.5 - 23.1] | 1.39 |
| BhQS11 | *Caballeronia udeis* LMG 27134 | 20.5 | [18.3 - 23.0] | 0.74 |
| BhQS11 | *Caballeronia sordidicola* LMG 22029 | 20.4 | [18.2 - 22.8] | 0.85 |

**Table S10.** DNA-DNA hybridization value prediction, with confidence intervals and GC% comparison, from Type (Strain) Genome Server version 2.1 (https://tygs.dsmz.de; accessed May 2019). The three new species of *Paraburkholderia* endosymbionts were compared against their closely related type strains, *Paraburkholderia* and *Caballeronia,* present on the server.
